# Glutathione-Responsive Fragmentation of Heteronorbornadiene-Based Thiovinyl Sulfones in Glioma Cells

**DOI:** 10.1021/acs.bioconjchem.5c00581

**Published:** 2026-04-28

**Authors:** Marina Carranza, Ana T. Carmona, Enrique Gil de Montes, Yanira Méndez, Inga Černauskienė, Gonçalo J. L. Bernardes, Antonio J. Moreno-Vargas

**Affiliations:** † Departamento de Química Orgánica, Facultad de Química, 16778 Universidad de Sevilla, C/Prof. García González, 1, Sevilla 41012, Spain; ‡ Yusuf Hamied Department of Chemistry, 2152 University of Cambridge, Lensfield Road, Cambridge CB2 1EW, U.K.; § Translational Chemical Biology Group, 16358 Spanish National Cancer Research Centre (CNIO), C/Melchor Fernández Almagro, 3., Madrid 28029, Spain

## Abstract

We report the synthesis and fluorescent properties of dansylated heteronorbornadienes (HNDs) featuring a thiovinyl sulfone moiety as a thiol-reactive trigger. These systems undergo thiol addition which promotes fragmentation via a thioketal intermediate, leading to the release of fluorescent heterocycles (furan or pyrrole) and ketene S,S-acetals. Distinct fluorescence signatures enable real-time monitoring of the fragmentation process. We also report cellular studies to demonstrate that HND-thiovinyl sulfones are membrane-permeable and undergo glutathione (GSH)-mediated activation in glioma cells, resulting in intracellular release of fluorescent products. These findings establish HND-based architectures as promising platforms for the design of cleavable linkers in controlled release applications, particularly in targeted drug delivery and theranostics where stimuli-responsive behavior is essential.

## Graphical abstract

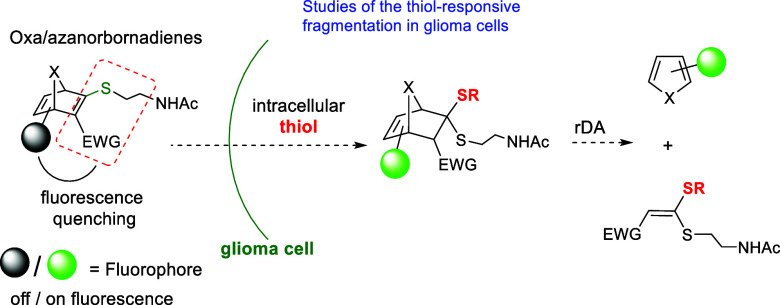

## Introduction

Glutathione (GSH) is a key component of the intracellular antioxidant defense system. It plays an essential role in maintaining cellular redox homeostasis and protecting against oxidative damage.^1^ Additionally, GSH is crucial for detoxifying xenobiotic electrophiles.^2^ Intracellular concentrations of GSH are particularly high in the cytosol, typically ranging from 1 to 10 mM, whereas its concentration in blood is markedly lower, between 2 and 10 μM.^3^ Notably, cancer cells exhibit even higher levels of GSH compared to normal cells, making GSH a valuable biomarker for tumor presence.^4^ Due to the pronounced difference between the intracellular and extracellular GSH concentration, this biothiol has emerged as an attractive stimulus in the development of drug delivery systems, enabling controlled intracellular drug release.^5,6^ Besides, GSH can act as an internal trigger to activate imaging agents, underscoring its dual role in both therapeutic strategies and molecular imaging in oncology.^6^

In the context of cleavable linkers, a widely adopted approach involves the use of GSH-sensitive disulfide bonds,^7,8^ which exploits the reductive nature of intracellular thiols, such as GSH, through thiol–disulfide exchange. However, disulfide-based conjugates often face stability issues in serum.^9^ This limitation is commonly addressed by introducing alkyl substituents near the disulfide linkage to provide steric hindrance against biothiol attack. Nonetheless, this steric protection can also hinder payload release inside cells, making it difficult to strike an optimal balance between serum stability and intracellular release efficiency.^10^ Therefore, there is a pressing need for the development of alternative GSH-sensitive linkers featuring different chemical functionalities and improved serum stability.

In this context, we have recently developed aza- and oxa-norbornadiene (AND and OND) systems functionalized with bromovinyl sulfone or ester moieties, capable of undergoing sequential thiol addition.^11^ We have shown that these systems enable site-selective bioconjugation to nanobodies at cysteine residues through a thiovinyl sulfone linkage. These bioconjugates exhibited high stability in the presence of human serum albumin (HSA) and plasma over several days, but they underwent GSH-triggered fragmentation via a tandem thiol-Michael addition/retro-Diels–Alder (rDA) sequence in the presence of an excess of GSH (Scheme 1).^11a,11c^

**Scheme 1:**
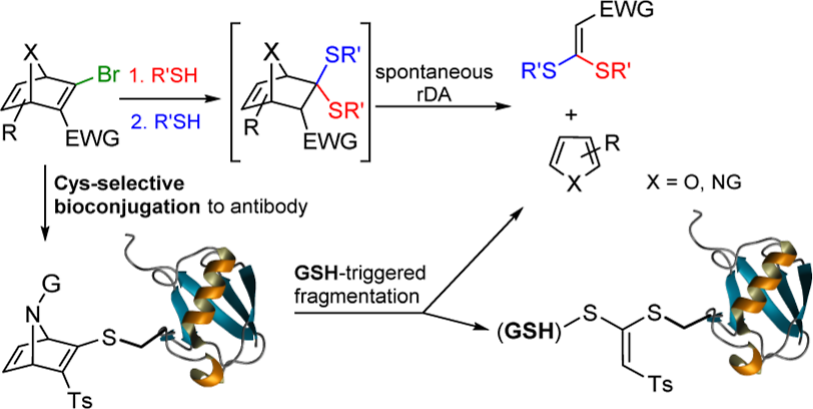
GSH-Promoted Fragmentation of AND-Bioconjugates

To verify that these heteronorbornadienic (HND) systems can be considered as promising thiol-sensitive linkers for drug delivery applications it is essential to demonstrate that the thiol-triggered fragmentation of model thiovinyl sulfones can indeed occur within the cytosolic environment of the cell, primarily mediated by GSH. For this purpose, in this work we have prepared a series of of dansylated heteronorbornadiene (HND) derivatives bearing a thiovinyl sulfone/ester moiety (Scheme 2) and studied their fluorescent properties. We expect an attenuation in fluorescence consistent with a donor-excited photoinduced electron transfer effect (d-PeT)^12^ from the fluorophore excited state to the LUMO of the electron-poor double bond system (thiovinyl (C_(2)_=C_(3)_) sulfone moiety), as previously observed by us in a related AND.^11c^ Structurally related oxanorbornadiene (OND) derivatives described by Finn and co-workers exhibited fluorescence modulation depending on the presence of an electron-deficient double bond adjacent to the fluorophore (dansyl).^13^ A similar quenching mechanism was extensively studied in maleimide–dansyl conjugate pairs.^14^ We hypothesize that dansyl-functionalized HNDs could undergo thiol-triggered fragmentation in the cytosol, releasing the corresponding fluorescent heterocycle and leading to detectable changes in fluorescence. Assuming that these compounds enter the cells via putative passive diffusion or other mediated uptake mechanisms, this fluorescence modulation would enable real-time monitoring of thiol-mediated cleavage events through confocal microscopy.

**Scheme 2:**
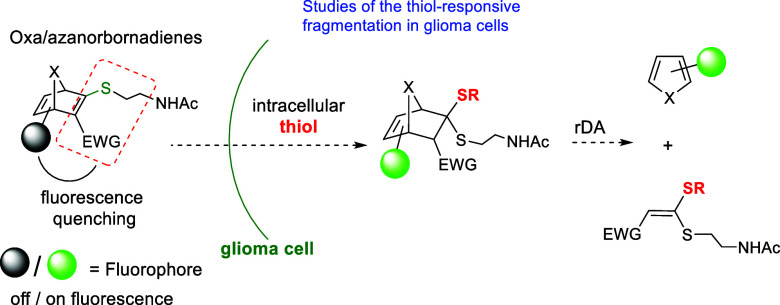
Proposed Thiol-Promoted Fragmentation of Dansylated HNDs Inside Cells

## Results and Discussion

### Synthesis and Reactivity of Dansylated HNDs toward Thiols

First, we prepared a batch of dansylated bromo-HNDs (**1**–**4**, Scheme 3) with different structural variations through a Diels–Alder reaction between a cyclic diene (furan or pyrrole derivative) and an electron-deficient bromoalkyne^15^ (for synthetic details see Supporting Information, Scheme S1): (1) O/NR in the bridge; (2) H/Me group at the bridgehead carbons C1/C4; (3) Ts/COOMe group at C3 as electron-withdrawing (EWG) group. The incorporation of the dansyl group at the N-bridge of bromo-AND **4** requires the addition of a glycine spacer. This strategy is based on our previous studies of thiol-promoted fragmentation in AND systems, which showed that acyl substituents on the bridging nitrogen accelerate fragmentation, whereas the sulfonamide group significantly prolongs fragmentation times (*t* > 3 days).^11c^

**Scheme 3:**
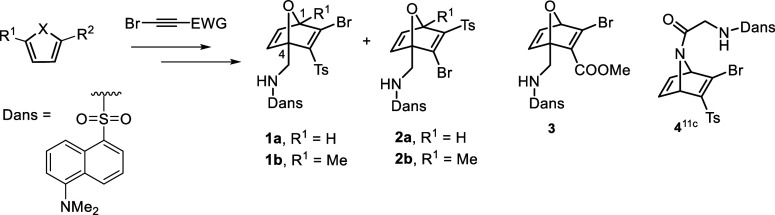
Synthesis of Dansylated Oxa- and Aza-Norbornadienes

Bromo-HNDs **1**–**4** were reacted with *N*-acetylcysteamine in MeCN (or DMF)/buffer (1.2:1, pH = 8) affording the desired thiovinyl sulfone/esters **5**–**10** in moderate-to-good yield (Scheme 4).

**Scheme 4:**
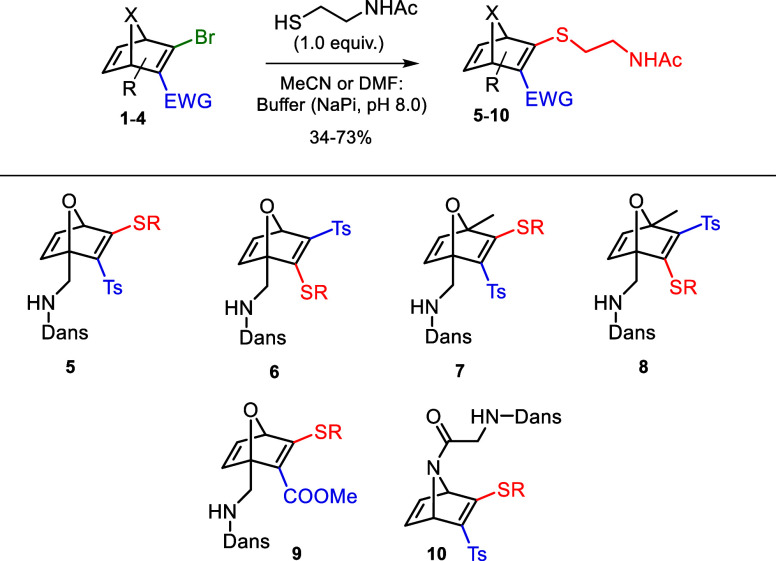
Synthesis of Dansylated Thio-HNDs

The subsequent thiol-promoted fragmentation of **5**–**10** was monitored by ^1^H NMR (Figures S1–S4). The reaction proceeds via a two-step mechanism: a thiol–Michael addition followed by a retro-Diels–Alder (rDA) reaction, affording a dansylated furan/pyrrole (**11**–**13**) and a ketene S,S-acetal (**14**–**15**). The parameter *t*, defined as the time required to reach 50% conversion of the starting HND into the corresponding furan/pyrrole product, was determined (Scheme 5). The measured *t* values varied from 70 min to 26 h.

**Scheme 5:**
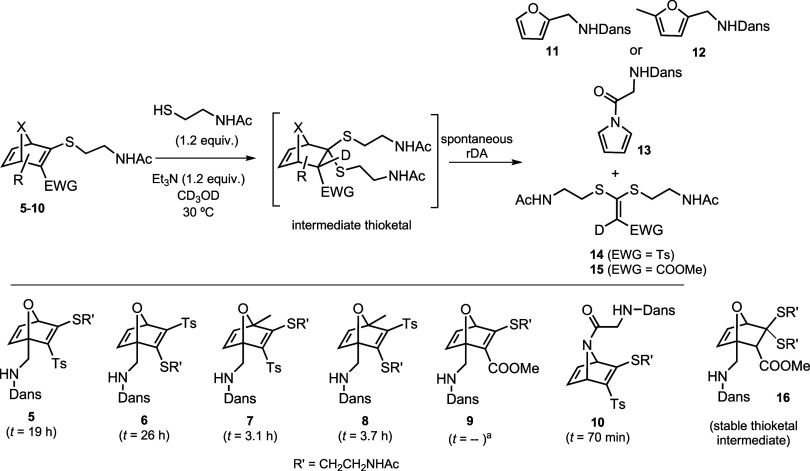
Thiol-Promoted Fragmentation of Dansylated Thio-HNDs and Time for the Transformation of Initial HND Into the 50% of the Expected Furan/Pyrrole (*t*)­^a^

a Final concentration of HND in the experiment: 0.068 M. rDA was the rate-limiting step in all the cases. ^a^Thioketal intermediate **16** was stable (no fragmentation was observed after 48 h)

All the HNDs originated rapidly (5–20 min) and almost quantitatively the corresponding thioketal intermediate (detected by ^1^H NMR), suggesting that the rDA is the rate-limiting step in all the cases. Thus, the comparative analysis of structural effects across this bicyclic series indicates that the observed differences arise exclusively from the rDA, rather than from the preceding conjugate addition. Both electronic and steric substituent effects appear to influence the observed rDA rates. Notably, replacement of the tosyl group at C3 with a methoxycarbonyl (**5** vs **9**) resulted in a dramatic decrease in the reaction rate, ultimately suppressing the rDA process under the examined conditions, allowing isolation of the stable thioketal intermediate **16**. This behavior can be rationalized by considering the stronger electron-withdrawing character of the tosyl group relative to the methoxycarbonyl. Increased electron withdrawal is expected to enhance the dienophilic character of the transient ketene *S*,*S*-acetal, thereby improving orbital interactions with the furanic diene, stabilizing the transition state of the rDA process, and consequently lowering the activation energy. Introduction of a methyl substituent at the bridgehead position led to a noticeable acceleration of the rDA reaction (**7** vs **5**), consistent with our previous observations in related oxanorbornene systems.^11b^ The methyl group likely increases the electron density of the developing furanic diene, which in turn strengthens the diene–dienophile interaction in the transition state. Finally, comparison of the oxabicyclic and azabicyclic systems (**5** vs **10**) reveals significantly faster fragmentation of the aza-analogue, likely influenced in part by steric effects. The thioketal intermediate derived from **10** exhibits greater steric congestion than its oxa-analogue **5** due to the bulky N-substituent. This increased steric demand accelerates the rDA by promoting strain relief along the pathway to the pyrrolic product. These results align with our previous studies, where aza-norbornenes consistently showed enhanced fragmentation rates compared to their oxa-counterparts.^11c^ These preliminary experiments showed that all the HNDs—with the exception of OND **9**—appear to meet the structural requirements to fragment under mild conditions.

### Fluorescence Studies

Dansylated-HNDs **5**–**10** showed an attenuated fluorescence with respect furan/pyrrole derivatives **11**–**13** (Figures 1 and S7–S13), presumably due to a PeT effect. Pyrrole **13** exhibited lower fluorescence than furans **11** and **12**, indicating that the acylated pyrrolic moiety partially quenches the fluorescence of the dansyl group. The fluorescence exhibited by the thioketal intermediate **16** indicates that the presence of the double bond between C2 and C3 in the HND systems seems to be necessary for a significant fluorescence quenching.

**Figure 1:**
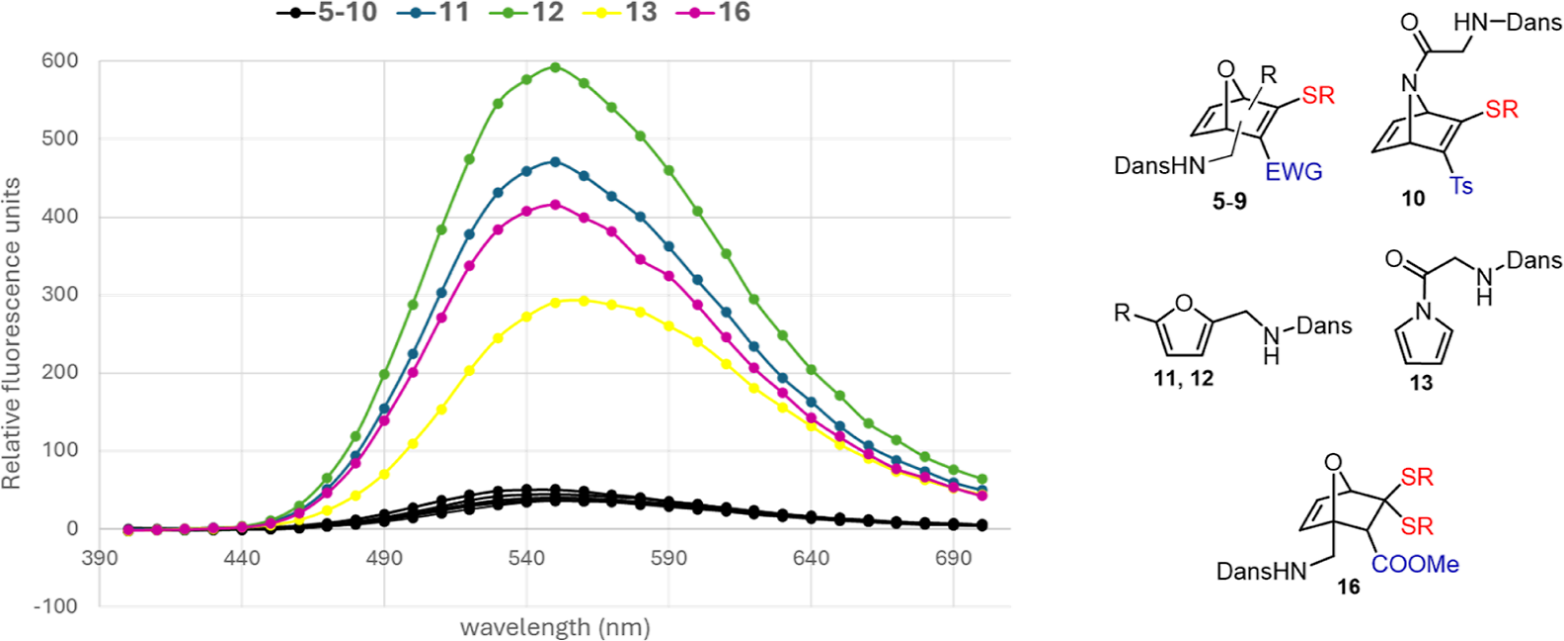
Emission plots (relative units) for dansylated HNDs and derivatives (λ_ex_ = 334 nm) at pH 7.4. A 1:1 Buffer (NaPi):DMSO solution of dansylated derivatives (100 μM) was used for the measurements. For individual plots, see Supporting Information (Section 3). *R* = CH_2_CH_2_NHAc.

The fragmentation of HNDs **5**–**8** and **10** was then evaluated in an aqueous medium containing an excess of GSH. The process was monitored by recording the fluorescence variation over a 5 h period (Figure 2).

**Figure 2:**
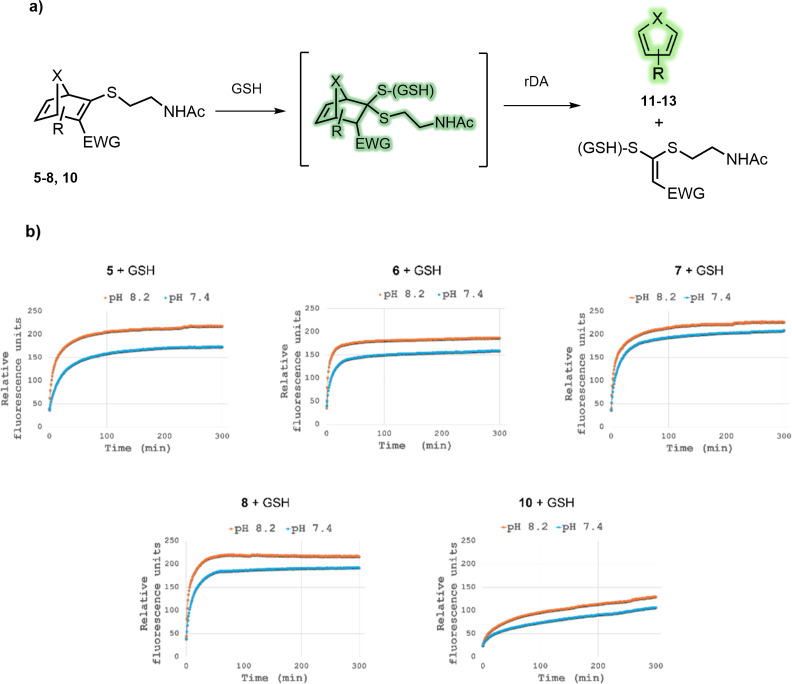
(a) General GSH-promoted fragmentation of dansylated thio-ONDs **5**–**8** and AND **10** (fluorescent structures are shown in green). (b) Fluorescence monitoring of the GSH-promoted fragmentation. Measurement conditions: To a 1:1 DMSO/buffered solution of HND (pH 8.2 or 7.4), GSH (1.5 equiv) in a buffered solution (pH 8.2 or 7.4) was added. The final concentration of HND in the experiment was 100 μM. Fluorescence measurements (λ_ex_ = 334 nm, λ_em_ = 550 nm) were recorded at 37 °C over a 5-h period.

All four ONDs **5**–**8** exhibited a rapid increase in fluorescence within the first few minutes following GSH addition, consistent with the immediate formation of the thioketal intermediate, in which the proposed quenching mechanism is largely inhibited, as previously confirmed for intermediate **16** (Figure 1). Monitoring the fragmentation of HNDs **5**–**8** and **10** in the presence of excess GSH revealed a moderate pH-dependent response. In all cases, both the initial rate of fluorescence enhancement and the maximum intensity were higher at pH 8.2 than at pH 7.4 (Figure 2). This observation is likely attributed to the higher concentration of the more nucleophilic thiolate species which facilitates the conjugate addition to the thiovinyl sulfone moiety. On the other hand, the difference in the shape of the fluorescence curves between ONDs (**5**–**8**) and AND **10** indicates a distinct behavior toward GSH. While the ONDs reached a plateau shortly after the initial fluorescence burst, the AND exhibited a sustained, gradual increase in emission over time. GSH-concentration dependent experiments of OND **5** and AND **10** produced a more pronounced fluorescence response in AND **10** compared to the oxa-analogue (See Supporting Information, Figures S14 and S15).

To gain further insight into the fragmentation of AND **10**, an additional experiment was performed under similar conditions but monitored by ^1^H NMR spectroscopy (see Supporting Information, Figure S5). The reaction was carried out in a DMSO-*d*_6_/D_2_O (1:1) mixture at pD 7.4, using AND **10** at 0.068 M and 2.0 equiv of GSH. The parameter *t* determined in this experiment was 187 min. The ^1^H NMR monitoring revealed signals corresponding only to the starting AND and the resulting dansylated pyrrole **13**, with no detectable thioketal intermediate. This outcome would be expected if the thiol–Michael addition were slower than the retro-Diels–Alder (rDA) reaction, which is consistent with the second-order kinetics determined for the fragmentation in this case (See Supporting Information, Figure S6). These results align with those obtained from fluorescence monitoring, where a gradual time-dependent fluorescence increase was observed.

^1^H NMR monitoring of the reaction of OND **1** in DMSO-*d*_6_/NaPi buffer (pD 7.4) was performed (Figure 3) to demonstrate the high efficiency of the transformation and the absence of detectable side-product formation. After 3 h, the spectra showed that most of the starting material had been converted into the thioketal intermediate and fragmentation products. Continued monitoring revealed a clean and progressive conversion of this intermediate into the fragmentation products, namely the furan derivative **11** and the S,S-ketene acetal, with complete consumption of the intermediate after 54 h. Importantly, no additional species were detected throughout the experiment, underscoring the high efficiency and remarkable cleanliness of the process.

**Figure 3:**
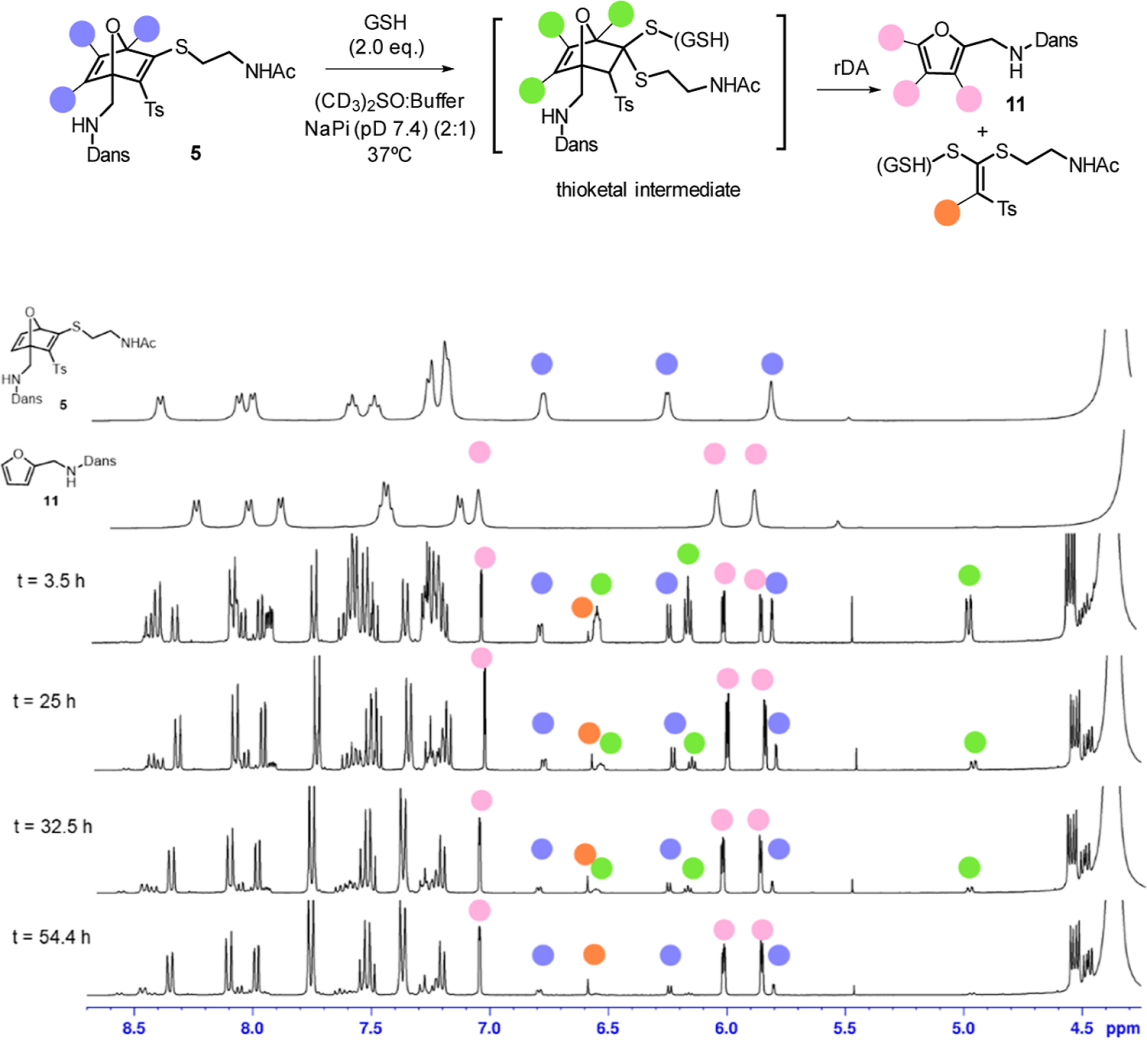
^1^H NMR-monitoring of the GSH-promoted fragmentation of thio-OND **5** in aqueous medium.

### Fragmentation of HNDs in Cells

Confocal fluorescence microscopy was employed to evaluate the cellular uptake and intracellular fragmentation of HNDs, taking advantage of the high intracellular concentration of GSH (2–10 mM) and the emission intensity changes that occur upon reaction of these systems with this biothiol. HNDs **5** and **10** were selected for the study in human glioma-derived cell line U87-MG.

Cells were cultured under standard conditions, and each well was treated with a final concentration of 1–20 μM of the corresponding HND. The samples were incubated at 37 °C and analyzed at two time points (2 and 4 h) to monitor the evolution of fluorescence associated with HND activation. To assess cellular internalization, membrane staining was performed with Germ Agglutinin–Alexa Fluor 647. Initial analysis of OND **5** revealed a gradual increase in green fluorescence within the red-stained cell boundaries, indicating efficient cellular internalization and subsequent reaction with intracellular GSH, resulting in activation of the dansyl group (Figures 4a and S16). This behavior is consistent with our previous observations: dansylated HNDs exhibit quenched fluorescence which is alleviated upon reaction with GSH and subsequent fragmentation. The increase in fluorescence intensity over time suggests successful conversion of the OND into fluorescent derivatives such as the thioketal intermediate and the final dansyl-furan product **11**. Upon incubation of AND **10** with U87-MG cells, this compound exhibited lower fluorescence intensity compared to OND **5** (Figures 4b and S17) under identical experimental conditions. This could be attributed to the lower fluorescence emission of the resulting dansyl-pyrrole **13** relative to the corresponding dansyl-furan **11**, as discussed in the previous section. To assess cellular localization, F-actin and nuclear staining were performed with Phalloidin-iFluor 647 and DAPI, respectively (Figure S18). Overlay images were expected to reveal the spatial localization of the fluorescent species relative to the cell membrane and nucleus. However, spectral overlap between the emission profiles of dansyl and DAPI complicated the interpretation of colocalization.

**Figure 4:**
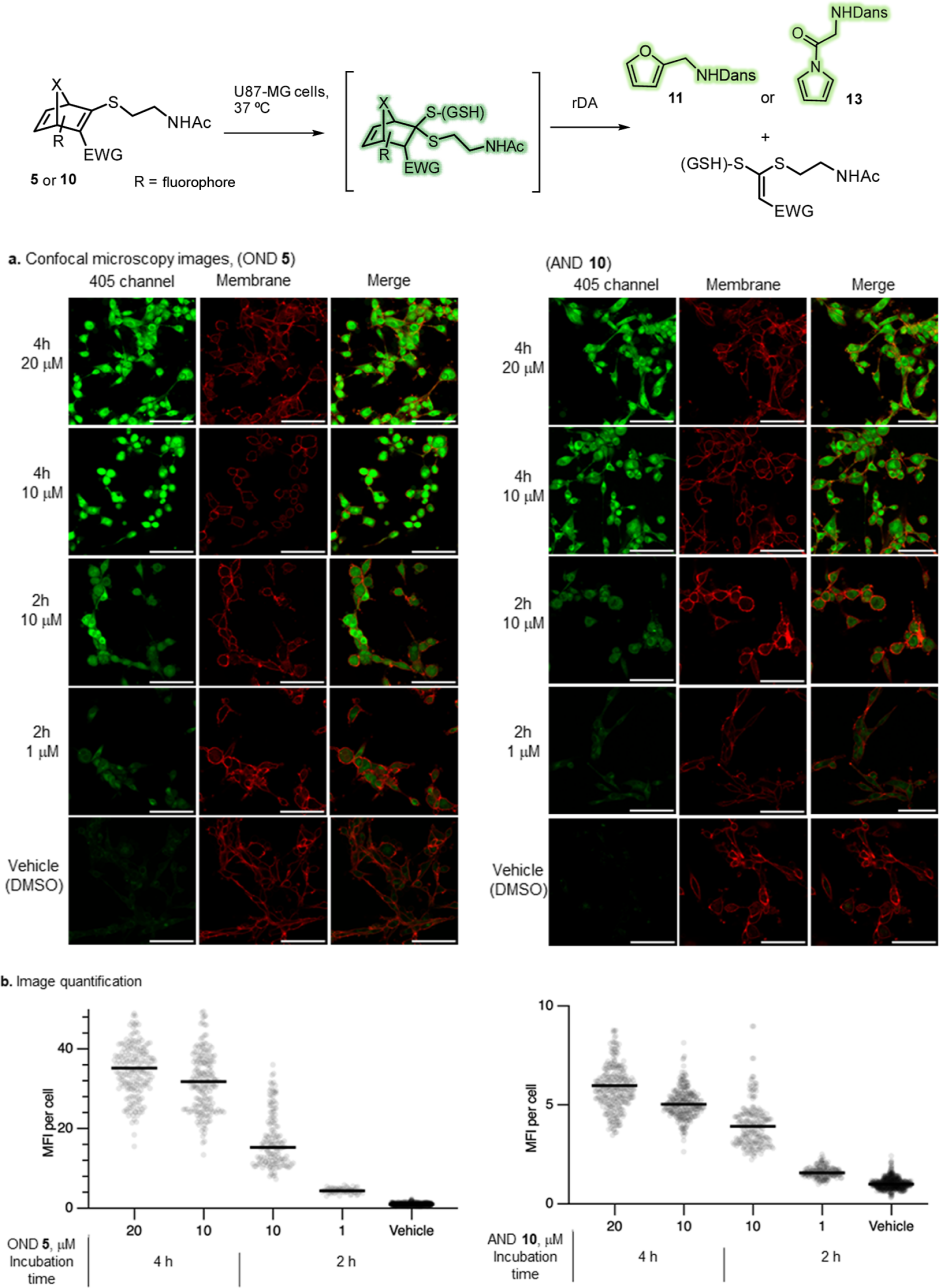
(a) Confocal microscopy images of U87-MG cells stained with Wheat Germ Agglutinin–Alexa Fluor 647 (cell membrane, red; λ_ex_ = 650 nm, λ_em_ = 665 nm) and after incubation with OND **5**/AND **10** (20 – 1 μM) for 4 or 2 h at 37 °C. The dansyl fluorescence corresponding to OND/AND activation is shown in green (λ_ex_ = 405 nm, λ_em_ = 550 nm). The white scale bar represents 100 μm. (b) Turn-on fluorescence quantification (image-wise ratio to DMSO vehicle control channel fluorescence) suggested significant OND **5**/AND **10** fluorescence in various concentrations. Each dot represents a single cell; data sets combine independent experiments.

To rule out the possibility that the fluorescence changes observed by confocal microscopy were influenced by compound-induced cell damage, the cytotoxicity of OND **5** and AND **10** was evaluated in glioma cells (see Supporting Information for details). Both compounds exhibited moderate-to-low cytotoxicity (IC_50_ ≈ 100 μM for AND **10**; IC_50_ = 36 μM for OND **5**, Figure S19) and were nontoxic up to 10 μM. Importantly, the fluorescence imaging response in cells at 10 μM was similar to that observed at 20 μM, indicating that the fluorescence changes are not attributable to cell damage.

## Conclusions

Fluorescence-quenched dansylated heteronorbornadienes (HNDs) bearing a thiovinyl sulfone functionality have been synthesized and shown to undergo a thiol-triggered reaction, leading to efficient fragmentation via a fluorescent thioketal intermediate. This cascade process results in the release of a fluorescent heterocyclic product (furan or pyrrole) and a ketene S,S-acetal. These dansyl-HNDs demonstrated good cell permeability and were activated by intracellular glutathione (GSH) in glioma cells, triggering the release of emissive products that could be monitored over time by confocal microscopy. These results highlight the potential of HND-based platforms as thiol-responsive cleavable linkers for controlled release applications, particularly in the design of targeted drug delivery systems or theranostic agents.

## Experimental Section

### General Methods

^1^H and ^13^C NMR spectra were recorded with a Bruker AVIII300, NEO300, NEO400, NEO500 and Spinsolve80 spectrometer for solutions in CDCl_3_, CD_3_OD, (CD_3_)_2_CO, (CD_3_)_2_SO, and C_6_D_6_. δ are given in ppm and *J* in Hz. Chemical shifts are calibrated using residual solvent signals. All the assignments were confirmed by 2D spectra (COSY and HSCQ). High resolution mass spectra were recorded on a Q-Exactive-quadrupole mass spectrometer. TLC was performed on silica gel 60 F_254_ (Merck), with detection by UV light charring with KMnO_4_, ninhydrin, or with reagent [(NH_4_)_6_MoO_4_, Ce­(SO_4_)_2_, H_2_SO_4_, H_2_O]. Purification by silica gel chromatography was carried out using either hand-packed glass columns (Silica gel 60 Merck, 40–60 and 63–200 μm) or Puriflash XS520 Plus Interchim system with prepacked cartridges. No unexpected or unusually high safety hazards were encountered.

### Synthesis of Thio-HNDs. General Procedure

To a solution of the corresponding dansyl bromo-HND (1.2 equiv) in DMF or MeCN (10 mL/mmol), a solution of *N*-acetylcysteamine (1.0 equiv) in DMF or MeCN (5 mL/mmol) and phosphate buffer solution (pH 8.0, 50 mM, 10 mL/mmol), were added simultaneously and the mixture was stirred at r.t. for 30 min. Then, solvents were evaporated, and the residue was dissolved in AcOEt and washed with water. The organic phase was dried with anh. Na_2_SO_4_, filtered and concentrated. Purification by silica gel column chromatography afforded the corresponding dansyl thio-HND.

#### (*rac*)-*N*-(2-((1-(((5-(Dimethylamino)­naphthalene)-1-sulfonamido)­methyl)-3-tosyl-7-oxabicyclo­[2.2.1]­hepta-2,5-dien-2-yl)­thio)­ethyl)­acetamide **(5)** and (*rac*)-*N*-(2-((4-(((5-(dimethylamino)­naphthalene)-1-sulfonamido)­methyl)-3-tosyl-7-oxabicyclo­[2.2.1]­hepta-2,5-dien-2-yl)­thio)­ethyl)­acetamide **(6)**

Starting from a mixture of **1a** and **2a** (500 mg, 0.85 mmol) and *N*-acetylcysteamine (92 mg, 0.77 mmol) in MeCN following the general procedure, afforded after chromatographic purification (EtOAc/Cy 10:1 → EtOAc) compound **5** (300 mg, 0.48 mmol, 62%, pale yellow solid) and **6** (48 mg, 0.08 mmol, 10%, yellow solid). Data for **5**: ^1^H NMR (300 MHz, CD_3_OD, 298 K, δ ppm): δ 8.55 (d, 1H, *J* = 8.5 Hz, Ar–H), 8.23 (d, 1H, *J* = 8.8 Hz, Ar–H), 8.09 (dd, 1H, *J* = 7.4, 1.1 Hz, Ar–H), 7.69 (d, 2H, *J* = 8.0 Hz, Ar–H), 7.59–7.50 (m, 2H, Ar–H), 7.31 (d, 2H, *J* = 8.0 Hz, Ar–H), 6.74 (dd, 1H, *J* = 5.7, 1.5 Hz, H-6), 6.59 (d, 1H, *J* = 5.4 Hz, H-5), 5.29 (d, 1H, *J* = 1.8 Hz, H-1), 3.53–3.48 (m, 2H, H-2′), 3.30 (s, 1H, CH_2_), 3.04 (s, 3H, H-1′, CH_2_), 2.76 s, 6H, (N­(CH_3_)_2_), 2.33 (s, 3H, CH_3_ of Ts), 1.80 (s, 3H, COCH_3_). ^13^C­{^1^H} NMR (75 MHz, CD_3_OD, 298 K, δ ppm): δ 172.3 (C=O), 171.4, 151.8 (C-2, C-3), 144.9 (*C*_q_Ar), 142.9 (C-5), 139.0 (C-6), 137.0, 136.2, 135.4, 129.8 (*C*_q_Ar), 129.6 (CH-Ar), 129.5 (*C*_q_Ar), 129.0, 127.9, 126.5, 122.9, 119.2, 115.1 (CH-Ar), 96.1 (C-4), 85.2 (C-1), 44.5 (N­(CH_3_)_2_), 41.9 (CH_2_), 40.3 (C-1′), 30.4 (C-2′), 20.2 (CH_3_ of Ts), 19.5 (COCH_3_). Data for **6**: ^1^H NMR (300 MHz, CD_3_OD, 298 K, δ ppm): δ 8.45 (d, 1H, *J* = 8.5 Hz, Ar–H), 8.23 (d, 1H, *J* = 8.6 Hz, Ar–H), 8.09 (dd, 1H, *J* = 8.5, 1.1 Hz, Ar–H), 7.59 (d, 2H, *J* = 8.0 Hz, Ar–H), 7.48–7.41 (m, 2H, Ar–H), 7.31 (d, 2H, *J* = 8.3 Hz, Ar–H), 7.15 (d, 1H, *J* = 7.5 Hz, Ar–H), 6.74 (dd, 1H, *J* = 5.4, 1.6 Hz, H-6), 6.59 (d, 1H, *J* = 5.2 Hz, H-6), 5.29 (d, 1H, *J* = 1.7 Hz, H-4), 3.64 (d, 1H, *J* = 14.6 Hz, CH_2_), 3.55 (d, 1H, *J* = 14.5 Hz, CH_2_), 3.04 (br. s, 4H, H-1′, H-2′), 2.87 (s, 6H, N­(CH_3_)_2_), 2.42 (s, 3H, CH_3_ of Ts), 1.90 (s, 3H, COCH_3_). ^13^C­{^1^H} NMR (75 MHz, CD_3_OD, 298 K, δ ppm): δ 171.9 (C=O), 161.9, 151.8 (C-2, C-3), 147.5, 145.1 (*C*_q_Ar), 144.2 (C-5), 139.7 (C-6), 136.9, 135.5 (*C*_q_Ar), 129.9 (CH-Ar), 129.8 (*C*_q_Ar), 129.6, 128.7, 127.8, 127.1, 122.9, 119.3, 115.1 (CH-Ar), 99.4 (C-1), 84.5 (C-4), 44.4 (N­(CH_3_)_2_), 41.6 (CH_2_), 39.7, 33.6 (C-1′, C-2′), 21.1 (COCH_3_), 20.2 (CH_3_ of Ts). HRESIMS *m*/*z*; found, 628.1589; calcd for C_30_H_34_N_3_O_6_S_3_ [M + H]^+^, 628.1604.

#### (*rac*)-*N*-(2-((1-(((5-(Dimethylamino)­naphthalene)-1-sulfonamido)­methyl)-4-methyl-3-tosyl-7-oxabicyclo­[2.2.1]­hepta-2,5-dien-2-yl)­thio)­ethyl)­acetamide **(7)** and (*rac*)-*N*-(2-((4-(((5-(dimethylamino)­naphthalene)-1-sulfonamido)­methyl)-1-methyl-3-tosyl-7-oxabicyclo­[2.2.1]­hepta-2,5-dien-2-yl)­thio)­ethyl)­acetamide **(8)**

Starting from a mixture of **1b** and **2b** (320 mg, 0.53 mmol) and *N*-acetylcysteamine (58 mg, 0.48 mmol) in MeCN following the general procedure, afforded after chromatographic purification (Et_2_O: MeOH 80:1→ 20:1) compound **7** (85 mg, 0.13 mmol, 27%, pale brown solid) and **8** (22 mg, 0.034 mmol, 7%, yellow solid). Data for **7**: ^1^H NMR (300 MHz, CDCl_3_, 298 K, δ ppm): δ 8.48 (d, 1H, *J* = 8.5, Ar–H), 8.20–8.16 (m, 2H, Ar–H), 7.55 (d, 2H, *J* = 7.2 Hz, Ar–H), 7.47 (d, 2H, *J* = 8.1 Hz, Ar–H), 7.20 (d, 2H, *J* = 7.3 Hz, Ar–H), 7.11 (d, 1H, *J* = 7.3 Hz, Ar–H), 6.54 (d, 1H, *J* = 5.0 Hz, H-5), 6.48 (d, 1H, *J* = 5.1 Hz, H-6), 6.43 (t, 1H, *J* = 5.4 Hz, NH), 5.33 (dd, 1H, *J* = 7.8, 4.5 Hz, CH_2_), 3.77 (dd, 1H, *J* = 14.0, 8.2 Hz, CH_2_), 3.41 (dd, 1H, *J =* 14.0, 4.5 Hz, CH_2_), 3.33–3.10 (m, 2H, H-2′), 2.97–2.86 (m, 2H, H-1′), 2.83 (s, 6H, N­(CH_3_)_2_), 2.34 (s, 3H, CH_3_ of Ts), 1.84 (s, 3H, COCH_3_), 1.53 (s, 3H, CH_3_). ^13^C­{^1^H} NMR (75 MHz, CDCl_3_, 298 K, δ ppm): δ 170.5 (C=O), 168.5, 152.0 (C-2, C-3), 150.9, 145.1 (*C*_q_Ar), 144.3 (C-5), 143.9 (C-6), 136.7, 134.8 (*C*_q_Ar), 130.5, 130.0, 129.9, 129.6, 128.5, 127.3, 123.2, 118.8, 115.3 (CH-Ar), 96.2, 94.8 (C-1, C-4), 45.4 (N­(CH_3_)_2_), 42.7 (CH_2_), 39.7 (C-2′), 33.3 (C-1′), 23.2 (COCH_3_), 21.7 (CH_3_ of Ts), 15.9 (CH_3_). Data for **8**: ^1^H NMR (300 MHz, CDCl_3_, 298 K, δ ppm): δ 8.55 (d, 1H, *J* = 7.5 Hz, Ar–H), 8.26–8.20 (m, 2H, Ar–H), 7.69 (d, 2H, *J* = 7.9 Hz, Ar–H), 7.56–7.49 (m, 2H, Ar–H), 7.33 (d, 2H, *J* = 7.4 Hz, Ar–H), 7.17 (d, 1H, *J* = 7.6 Hz, Ar–H), 6.69–6.59 (m, 3H, H-5, H-6, NH), 5.11 (t, 1H, *J* = 6.1 Hz, NH), 3.77 (d, 2H, *J* = 6.0 Hz, CH_2_), 3.44–3.34 (m, 1H, H-2′), 3.28–3.17 (m, 1H, H-2′), 3.00 (t, 2H, *J* = 6.1 Hz, H-1′), 2.88 (s, 6H, N­(CH_3_)_2_), 2.42 (s, 3H, CH_3_ of Ts), 1.95 (s, 3H, COCH_3_), 1.62 (s, 3H, CH_3_). ^13^C­{^1^H} NMR (75 MHz, CDCl_3_, 298 K, δ ppm): δ 170.5 (C=O), 168.5, 152.0 (C-2, C-3), 150.9, 145.1 (*C*_q_Ar), 144.3 (C-5), 144.0 (C-6), 136.7, 134.8 (*C*_q_Ar), 130.5, 130.0, 129.9, (CH-Ar), 129.6 (*C*_q_Ar), 128.5, 127.3, 123.2, 118.8, 115.3 (CH-Ar), 96.2, 94.8 (C-1, C-4), 45.5 (N­(CH_3_)_2_), 42.7 (CH_2_), 39.7, 33.5 (C-1′, C-2′), 23.2 (COCH_3_), 21.7 (CH_3_ of Ts), 15.9 (CH_3_). HRESIMS *m*/*z*; found, 642.1758; calcd for C_31_H_36_N_3_O_6_S_3_ [M + H]^+^, 642.1761.

#### (*rac*)-Methyl-3-((2-acetamidoethyl)­thio)-1-(((5-(dimethylamino)­naphthalene)-1-sulfonamido)­methyl)-7-oxabicyclo­[2.2.1]­hepta-2,5-diene-2-carboxylate **(9)**

Starting from **3** (118 mg, 0.24 mmol) and *N*-acetylcysteamine (26 mg, 0.22 mmol) in DMF following the general procedure, afforded after chromatographic purification (EtOAc/Cy 10:1→ EtOAc) compound **9** (85 mg, 0.16 mmol, 73%, pale yellow solid). ^1^H NMR (300 MHz, CD_3_OD, 298 K, δ ppm): δ 8.56 (d, 1H, *J* = 8.8 Hz, Ar–H), 8.35 (d, 1H, *J* = 8.7 Hz, Ar–H), 8.25 (dd, 1H, *J* = 7.5, 1.3 Hz, Ar–H), 7.61–7.54 (m, 2H, Ar–H), 7.27 (d, 1H, *J* = 7.5 Hz, Ar–H), 7.05 (dd, 1H, *J* = 5.3, 2.0 Hz, H-5), 6.82 (d, 1H, *J* = 5.3 Hz, H-6), 5.77 (d, 1H, *J* = 1.9 Hz, H-4), 3.96 (d, 1H, *J* = 14.5 Hz, CH_2_), 3.67 (d, 1H, *J* = 14.4 Hz, CH_2_), 3.52 (s, 3H, COOCH_3_), 3.36–3.32 (m, 2H, H-2′), 3.15–3.03 (m, 2H, H-1′), 2.88 (s, 6H, N­(CH_3_)_2_), 1.94 (s, 3H, COCH_3_). ^13^C­{^1^H} NMR (75 MHz, CD_3_OD, 298 K, δ ppm): δ 173.7 (C=O), 172.3 (C=O), 164.2, 163.5 (C-2, C-3), 151.8 (*C*_q_Ar), 143.6 (C-6), 141.9 (C-5), 135.7, 131.0, 129.8 (*C*_q_Ar), 129.7, 128.8, 127.2, 122.9, 119.3, 115.0 (CH-Ar), 95.4 (C-1), 84.2 (C-4), 50.3 (COOCH_3_), 44.5 (N­(CH_3_)_2_), 42.3 (CH_2_), 40.4 (C-2′), 30.3 (C-1′), 21.1 (COCH_3_). HRESIMS *m*/*z*; found, 532.1564; calcd for C_25_H_30_N_3_O_6_S_2_ [M + H]^+^, 532.1517.

#### (*rac*)-*N*-(2-((7-(((5-(Dimethylamino)­naphthalen-1-yl)­sulfonyl)­glycyl)-3-tosyl-7-azabicyclo­[2.2.1]­hepta-2,5-dien-2-yl)­thio)­ethyl)­acetamide **(10)**

Starting from **4** (82 mg, 0.13 mmol) and *N*-acetylcysteamine (14 mg, 0.12 mmol) in MeCN following the general procedure, afforded after chromatographic purification (EtOAc/Cy 10:1→ EtOAc) compound **10** (40 mg, 0.061 mmol, 51%, pale yellow solid). ^1^H NMR (300 MHz, CD_3_OD, 298 K, δ ppm, mixture of rotamers): δ 8.57 (d, 1H, *J* = 8.4 Hz, Ar–H), 8.33 (d, 1H, *J* = 8.6 Hz, Ar–H), 8.15 (dd, 1H, *J* = 7.5 Hz, 1.1 Hz, Ar–H), 7.69 (d, 1H, *J* = 8.3 Hz, Ar–H), 7.63–7.54 (m, 2H, Ar–H), 7.41–7.34 (m, 2H, Ar–H), 7.28 (d, 1H, *J* = 7.6 Hz, Ar–H), 6.96–6.67 (m, 2H, H-5, H-6), 5.85 (s, 1H, H-1 or H-4), 5.54 (s, 1H, H-1 or H-4), 3.81–3.51 (m, 2H, H-2′), 3.27–3.02 (m, 2H, H-1′), 2.89 (s, 3H, N­(CH_3_)_2_), 2.38 (s, 3H, CH_3_ of Ts), 1.94 (s, 3H, COCH_3_). ^13^C­{^1^H} NMR (75 MHz, CD_3_OD, 298 K, δ ppm, mixture of rotamers): δ 173.6, 170.3, 167.2, 164.4, 153.2, 146.6, 144.5, 143.7, 140.4, 139.0, 137.9, 136.8, 131.3, 131.2, 131.1, 1230.1, 129.9, 129.1, 128.2, 124.4, 120.7, 116.7, 69.7, 56.2, 45.8, 41.3, 32.4, 22.5, 22.6. HRESIMS *m*/*z*; found, 655.1706; calcd for C_31_H_35_N_4_O_6_S_3_ [M + H]^+^, 655.1732.

#### (*rac*)-Methyl 3,3-bis­((2-acetamidoethyl)­thio)-1-(((5-(dimethylamino)­naphthalene)-1-sulfonamido)­methyl)-7-oxabicyclo[2.2.1]­hept-5-ene-2-*endo*-carboxylate **(16)**

To a solution of **9** (22 mg, 0.041 mmol) in CD_3_OD (0.5 mL), *N*-acetylcysteamine (6.0 mg, 0.050 mmol) and Et_3_N (7 μL, 0.05 mmol) in CD_3_OD (0.1 mL) were added and the reaction mixture was stirred at 30 °C for 2 days. Then, the solvent was removed, and the residue was purified by column chromatography on silica gel (DCM: MeOH 20:1) to afford **16** (11 mg, 0.017 mmol, 41%, yellow solid). ^1^H NMR (300 MHz, CD_3_OD, 298 K, δ ppm): δ 8.47 (d, 1H, *J* = 8.6 Hz, Ar–H), 8.25 (d, 1H, *J* = 8.9 Hz, Ar–H), 8.06 (dd, 1H, *J* = 7.4 Hz, 1.3 Hz, Ar–H), 7.52–7.42 (m, 2H, Ar–H), 7.18 (d, 1H, *J* = 7.7 Hz, Ar–H), 6.31 (dd, 1H, *J* = 6.0, 1.9 Hz, H-5), 6.21 (d, 1H, *J* = 5.7 Hz, H-6), 4.70 (d, 1H, *J* = 1.9 Hz, H-4), 3.40 (s, 3H, COOCH_3_), 3.38–3.30 (m, 4H, H-2′), 2.80 (s, 6H, N­(CH_3_)_2_), 2.77–2.63 (m, 4H, H-1′), 1.84 (s, 3H, COCH_3_), 1.80 (s, 3H, COCH_3_). ^13^C­{^1^H} NMR (75 MHz, CD_3_OD, 298 K, δ ppm): δ 172.1, 171.94, 171.89, 169.1, 151.8, 136.4, 135.5, 135.2, 130.0, 129.9, 129.5, 128.8, 127.9, 122.9, 119.2, 115.1, 90.9, 86.8, 68.5, 50.9, 44.4, 42.6, 39.0, 38.6, 38.4, 38.3, 37.0, 30.7, 29.8, 21.2, 21.1. HRESIMS *m*/*z*; found, 673.1778; calcd for C_29_H_38_N_4_O_7_NaS_3_ [M + H]^+^, 673.1795.

## Supplementary Material



## Notes

Synthesis of starting materials, NMR-monitorization of the thiol-promoted fragmentation of HNDs, fluorescence emission plots, details for cell imaging experiments and cell viability assays, ^1^H and ^13^C NMR spectra for new compounds (PDF)
