# Supplementary material for: Glutathione-Responsive Fragmentation of Heteronorbornadiene-Based Thiovinyl Sulfones in Glioma Cells
Source: Bioconjug Chem. 2026 Apr 28;37(5):906–14. doi: 10.1021/acs.bioconjchem.5c00581 (PMC13195567; doi:10.1021/acs.bioconjchem.5c00581)
Supplement: Supplementary file 1 [file bc5c00581_si_001.pdf]

SUPPORTING INFORMATION

Glutathione-responsive fragmentation of heteronorbornadiene-based thiovinyl sulfones in glioma cells

Marina Carranza,[a] Ana T. Carmona,[a,*] Enrique Gil de Montes,[b] Yanira Méndez,[b] Inga Černauskienė,[b] Gonçalo J. L. Bernardes[b,c,*] and Antonio J. Moreno-Vargas[a,*]

[a]*Departamento de Química Orgánica, Facultad de Química, Universidad de Sevilla, C/ Prof. García González, 1, 41012-Sevilla, Spain. E-mails: anatere@us.es, ajmoreno@us.es*

[b]*Yusuf Hamied Department of Chemistry, University of Cambridge, Lensfield Road, CB2 1EW Cambridge, UK. E-mail: gb453@cam.ac.uk*

[c]*Translational Chemical Biology Group, Spanish National Cancer Research Centre (CNIO), C/ Melchor Fernández Almagro, 3. 28029 Madrid, Spain*

TABLE OF CONTENTS

1. Synthesis of dansyl bromo-HNDs S2

2. ^{1}H NMR monitoring of the fragmentation reaction of dansyl-HNDs. S5

3. Fluorescence experiments S11

4. Cell imaging experiments S17

5. Cell viability assays S20

6. ^{1}H and ^{13}C-NMR spectra for new compounds S21

7. References S32

1. Synthesis of dansyl bromo-HNDs.

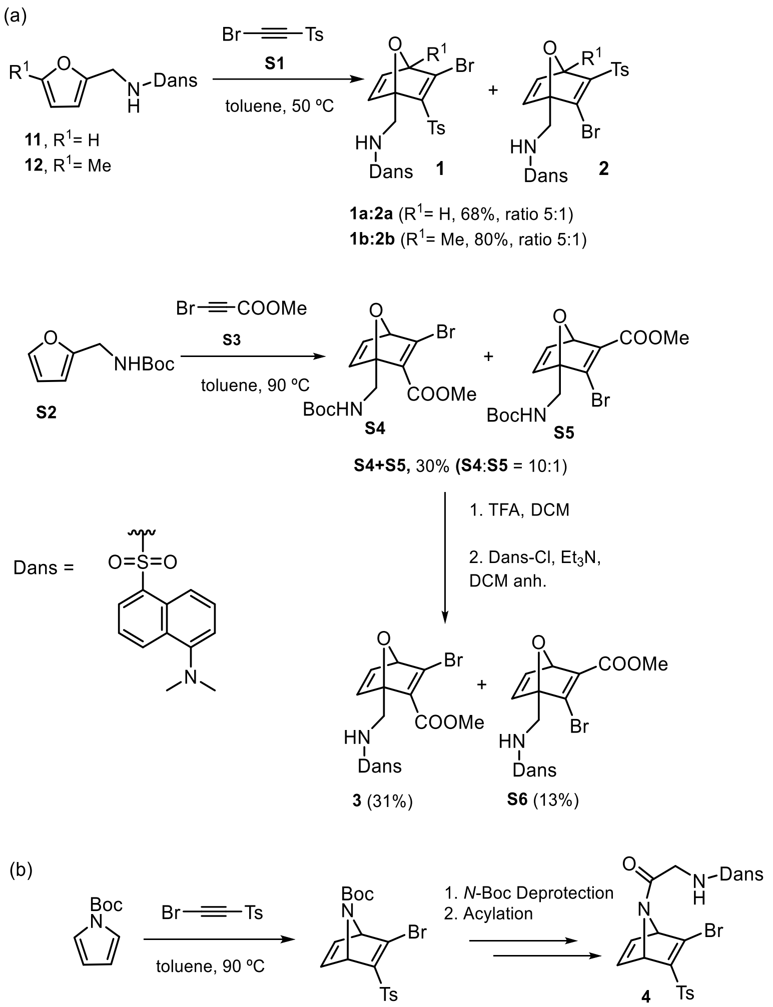

Scheme S1. Synthesis of dansyl (a) oxanorbornadienes (**1**-**3**) and (b) aza-norbornadiene (**4**). For the synthesis of **11** and **12**, see ref. 1; for the synthesis of **S1**, see ref. 2; for the synthesis of AND **4**, see ref. 3.

(*rac*)-*N*-((-3-Bromo-2-tosyl-7-oxabicyclo[2.2.1]hepta-2,5-dien-1-yl)methyl)-5-(dimethylamino)naphthalene-1-sulfonamide (1a) and (*rac*)-*N*-((-2-bromo-3-tosyl-7-oxabicyclo[2.2.1]hepta-2,5-dien-1-yl)methyl)-5-(dimethylamino)naphthalene-1-sulfonamide (2a).

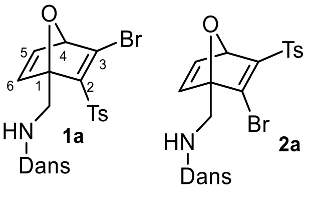

To a solution of **11** (493 mg, 1.49 mmol) in toluene (4 mL), alkyne **S1** (720 mg, 2.77 mmol) was added, and the mixture was stirred at 50 °C for 22 h. Then, the solvent was evaporated, and the

resulting residue was purified by column chromatography (EtOAc:CyH 1:8) to afford a mixture of **1a** and **2a** (600 mg, 1.01 mmol, 68%, pale-yellow solid, regioisomer ratio 5:1). Data for **1a** (major regioisomer): ^{1}H NMR (300 MHz, $CDCl_3$, 298 K, δ ppm): δ 8.58 (d, 1H, *J* = 8.8 Hz, Ar-H), 8.20-8.17 (m, 2H, Ar-H), 7.55-7.43 (m, 4H, Ar-H), 7.22-7.19 (m, 2H, Ar-H), 7.12 (d, 1H, *J* = 7.4 Hz, Ar-H), 6.78 (dd, 1H, *J* = 5.5, 2.0 Hz, H-5), 6.60 (d, 1H, *J* = 5.5 Hz, H-6), 5.43-5.39 (m, 1H, NH), 5.10 (d, 1H, *J* = 2.0 Hz, H-4), 3.81 (dd, 1H, *J* = 16.5, 8.6 Hz, CH_2), 3.43 (dd, 1H, *J* = 15.0, 4.0 Hz, CH_2), 2.83 (s, 6H, $N(CH_3)_2$), 2.35 (s, 3H, CH_3 of Ts). $^{13}C\{^1H\}$ NMR (75 MHz, $CDCl_3$, 298 K, δ ppm): δ 149.3, 149.2 (C-2, C-3), 145.6, 145.0 (C_qAr), 143.6 (C-6), 140.5 (C-5), 135.3, 134.8 (C_qAr), 130.6, (CH-Ar), 130.1 (C_qAr), 129.9, 129.5, 128.6, 127.8 (CH-Ar), 127.6 (C_qAr), 123.2, 118.7, 115.4 (CH-Ar), 96.6 (C-1), 89.2 (C-4), 45.4 ($N(CH_3)_2$), 42.4 (CH_2), 21.8 (CH_3 of Ts). HRESIMS m/z: found, 589.0452; calcd. for $C_{26}H_{26}{}^{79}BrN_2O_5S_2$ $[M+H]^+$, 589.0462.

(*rac*)-*N*-((-3-Bromo-4-methyl-2-tosyl-7-oxabicyclo[2.2.1]hepta-2,5-dien-1-yl)methyl)-5-(dimethylamino)naphthalene-1-sulfonamide (1b) and (*rac*)-*N*-((-2-bromo-4-methyl-3-tosyl-7-oxabicyclo[2.2.1]hepta-2,5-dien-1-yl)methyl)-5-(dimethylamino)naphthalene-1-sulfonamide (2b).

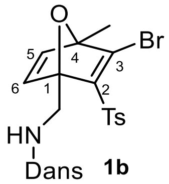

1b

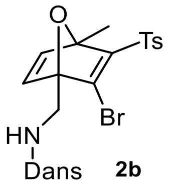

2b

To a solution of **12** (210 mg, 0.61 mmol) in toluene (3 mL), alkyne **S1** (236 mg, 0.91 mmol) was added and the mixture was stirred at 50 °C for 2 days. Then, the solvent was evaporated, and the resulting residue was purified by column chromatography (Et_2O:Cy 1:4) to afford a mixture of **1b** and **2b** (296 mg, 0.49 mmol, 80%, pale-yellow solid, ratio 5:1). Data for **1b** (major regioisomer): ^{1}H NMR (300 MHz, $CDCl_3$, 298 K, δ ppm): δ 8.56 (d, 1H, *J* = 8.5 Hz, Ar-H), 8.29-8.23 (m, 2H, Ar-H), 7.70-7.49 (m, 4H, Ar-H), 7.35–7.17 (m, 3H, Ar-H), 6.61 (d, 1H, *J* = 5.2 Hz, H-5), 6.54 (d, 1H, *J* = 5.3 Hz, H-6), 5.54–5.49 (m, 1H, NH), 3.87 (dd, 1H, *J* = 14.6, 8.6 Hz, CH_2), 3.49 (dd, 1H, *J* = 14.3, 4.5 Hz, CH_2), 2.90 (s, 3H, $N(C\underline{H_3})_2$), 2.42 (s, 3H, CH_3 of Ts), 1.52 (s, 3H, CH_3). $^{13}C\{^1H\}$ NMR (75 MHz, $CDCl_3$, 298 K, δ ppm): δ 153.4, 150.0, 148.0, 145.5 (2C_qAr, C-2 or C-3), 144.4 (C-5), 143.7 (C-6), 140.9, 135.3 135.0 (2C_qAr, C-2 or C-3), 130.5, 130.0, 129.9, 128.5, (CH-Ar), 127.7 (C_qAr), 127.6, 123.2 118.6, 115.4 (CH-Ar), 95.6, 95.0 (C-1, C-4), 45.5 ($N(\underline{C}H_3)_2$), 42.6 (CH_2), 21.7 (CH_3 of Ts), 15.2 (CH_3). HRESIMS m/z: found, 589.0452; calcd. for $C_{27}H_{28}{}^{79}BrN_2O_5S_2$ $[M+H]^+$: 589.0461.

(*rac*)- Methyl-3-bromo-1-(((*tert*-butoxycarbonyl)amino)methyl)-7-oxabicyclo-[2.2.1]hepta-2,5-diene-2-carboxylate (S4) and (*rac*)-Methyl-3-bromo-4-(((*tert*-butoxycarbonyl)amino)methyl)-7-oxabicyclo[2.2.1]hepta-2,5-diene-2-carboxylate (S5).

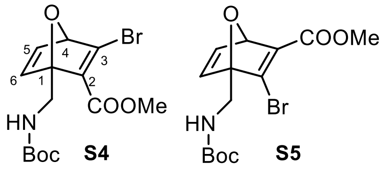

To a solution of **S2** (600 mg, 3.04 mmol) in toluene (12 mL), commercial alkyne **S3** (1.48 g, 9.14 mmol) was added, and the mixture was stirred at 90 °C overnight in a sealed tube. Then, the solvent was evaporated, and the resulting residue was purified by column chromatography (EtOAc:Cy 1:6) to afford a mixture of **S4** and **S5** (324 mg, 0.90 mmol, 30%, brown oil, regioisomer ratio 10:1). Data for **S4** (major regioisomer): ^{1}H NMR (300 MHz, $CDCl_3$, 298 K, δ ppm): δ 7.16 (dd, 1H, *J* = 5.2. 1.8 Hz, H-5), 7.03 (d, 1H, *J* = 5.2 Hz, H-6), 5.25 (d, 1H, *J* = 1.7 Hz, H-4), 4.96 (br. s, 1H, NH), 4.15 (dd, 1H, *J* = 14.4, 6.3 Hz, CH_2), 3.93 (dd, 1H, *J* = 14.3, 6.1 Hz, CH_2), 3.79 (s, 3H, $COOC\underline{H_3}$), 1.43 (s, 9H, CH_3 of Boc). ^{13}C{^{1}H} NMR (75 MHz, $CDCl_3$, 298 K, δ ppm): δ 162.9 (C=O), 155.8 (C=O), 151.0 (C-2 or C-3), 144.1 (C-6), 142.8 (C-5), 142.2 (C-2 or C-3), 96.7 (C-1), 88.4 (C-4), 79.6 (C_q of Boc), 51.8 ($COO\underline{C}H_3$), 39.9 (CH_2), 26.9 (CH_3 of Boc). HRESIMS m/z: found, 382.0262; calcd. for $C_{14}H_{18}{}^{79}BrNO_5Na$ $[M+Na]^+$, 382.0266.

(*rac*)-Methyl-3-bromo-1-(((5-(dimethylamino)naphthalene)-1-sulfonamido)methyl)-7-oxabicyclo[2.2.1]hepta-2,5-diene-2-carboxylate (3) and (*rac*)-Methyl-3-bromo-4-(((5-(dimethylamino)naphthalene)-1-sulfonamido)methyl)-7-oxabicyclo[2.2.1]hepta-2,5-diene-2-carboxylate (S6).

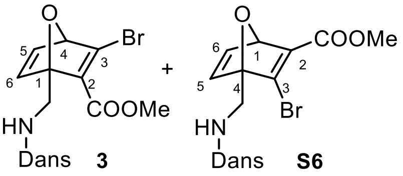

To a solution of **S4** and **S5** (300 mg, 0.84 mmol) in DCM (10 mL), TFA (2.0 mL, 25 mmol) was added. The reaction was stirred at r.t. for 30 min and then, the solvent was removed co-evaporating with toluene. The resulting product (0.84 mmol) was dissolved in anh. DCM (15 mL) and dansyl chloride (450 mg, 1.68 mmol), and Et_3N (240 µL, 1.68 mmol) were added. The mixture was stirred at r.t. for 1 h and then, it was diluted with DCM and washed with H_2O. The organic phase was separated, dried with anh. Na_2SO_4, filtered and concentrated. The residue was purified by column chromatography on silica gel (EtOAc:Cy 1:3) to afford **3** (130 mg, 0.26 mmol,

31% two steps, yellow solid), and **S6** (52 mg, 0.11 mmol, 13% two steps, yellow solid). Data for **3**: ^{1}H NMR (300 MHz, $CDCl_3$, 298 K, δ ppm): δ 8.54 (d, 1H, *J* = 8.6 Hz, Ar-H), 8.28-8.23 (m, 2H, Ar-H), 7.63-7.50 (m, 2H, Ar-H), 7.18 (d, 1H, *J* = 7.0 Hz, Ar-H), 7.07 (dd, 1H, *J* = 5.3 Hz, 1.8 Hz, H-5), 6.90 (d, 1H, *J* = 5.5 Hz, H-6), 5.37 (t, 1H, *J* = 6.3 Hz, NH), 5.16 (d, 1H, *J* = 1.9 Hz, H-4), 3.84-3.67 (m, 2H, CH_2), 3.63 (s, 3H, $COOC\underline{H_3}$), 2.87 (s, 6H, $N(CH_3)_2$). $^{13}C\{^{1}H\}$ NMR (75 MHz, $CDCl_3$, 298 K, δ ppm): δ 163.0 (C=O), 152.0, 150.9 (C-2, C-3), 143.9 (C-6), 142.5 (C-5), 141.8, 134.5, 130.6 (C_qAr), 129.9, 129.8 (CH-Ar), 129.7 (C_qAr), 128.5, 123.2, 118.8, 115.3 (CH-Ar), 95.5 (C-1), 88.4 (C-4), 51.8 ($COO\underline{C}H_3$), 45.4 ($N(\underline{C}H_3)_2$), 42.5 (CH_2). HRESIMS m/z: found, 493.0433; calcd. for $C_{21}H_{22}{}^{79}BrN_2O_5S$ $[M+H]^+$, 493.0427.

2. ^{1}H NMR monitoring of the fragmentation reaction of dansyl-HNDs.

2.1 General procedure for kinetic experiments *via* ^{1}H NMR.

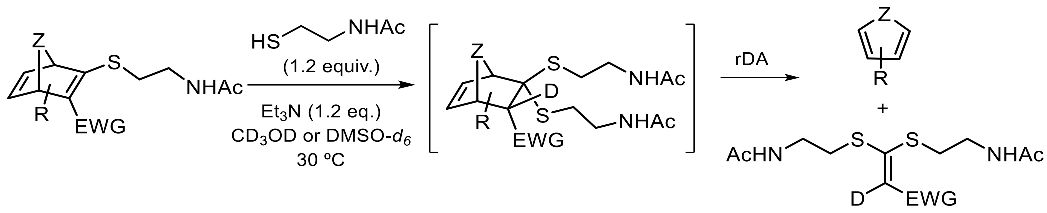

The corresponding HND (0.04 mmol) was dissolved in CD_3OD or DMSO-d_6 (0.5 mL) in a NMR tube. Then, *N*-acetylcysteamine (0.05 mmol) and triethylamine (0.05 mmol) in CD_3OD or DMSO-d_6 (0.1 mL) were added, and the reaction was left to complete at 30 °C. ^{1}H-NMR spectra were registered at regular intervals.

2.2 ^{1}H NMR monitoring of fragmentation reaction of dansyl-HNDs.

Compound 5

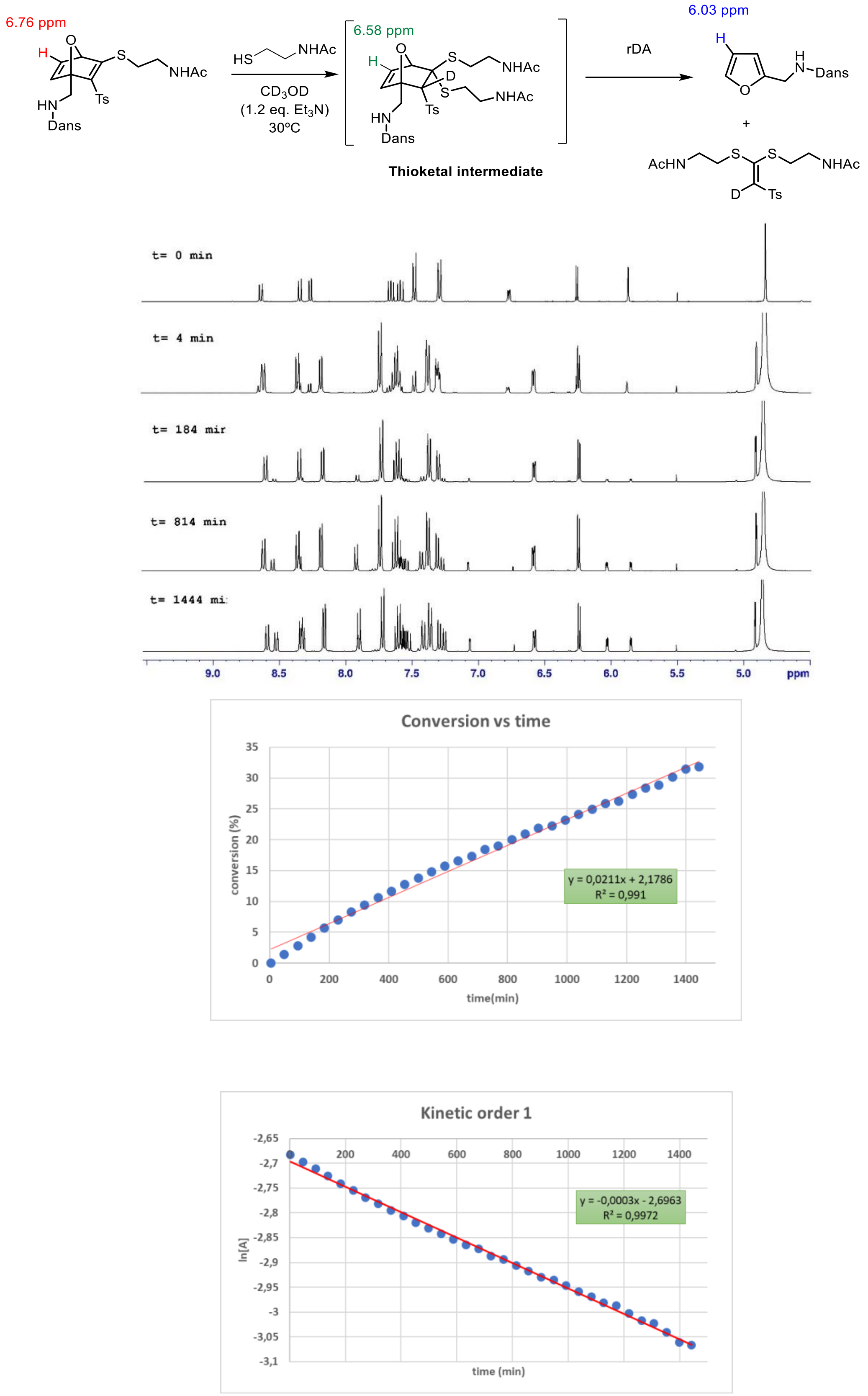

Figure S1. ^{1}H-NMR monitoring of the thiol-promoted fragmentation of thio-OND **5**. Determination of the kinetic order.

Compound 7

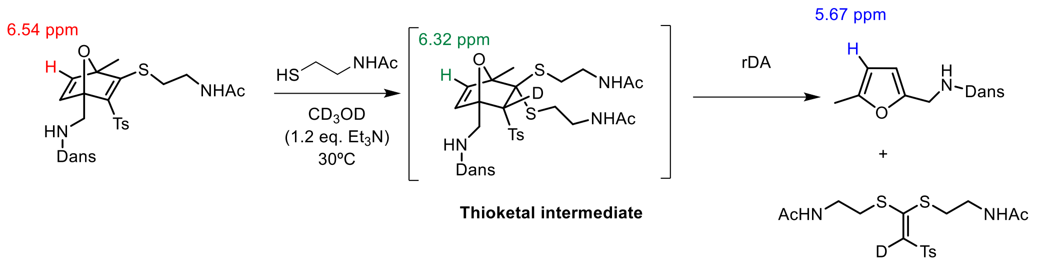

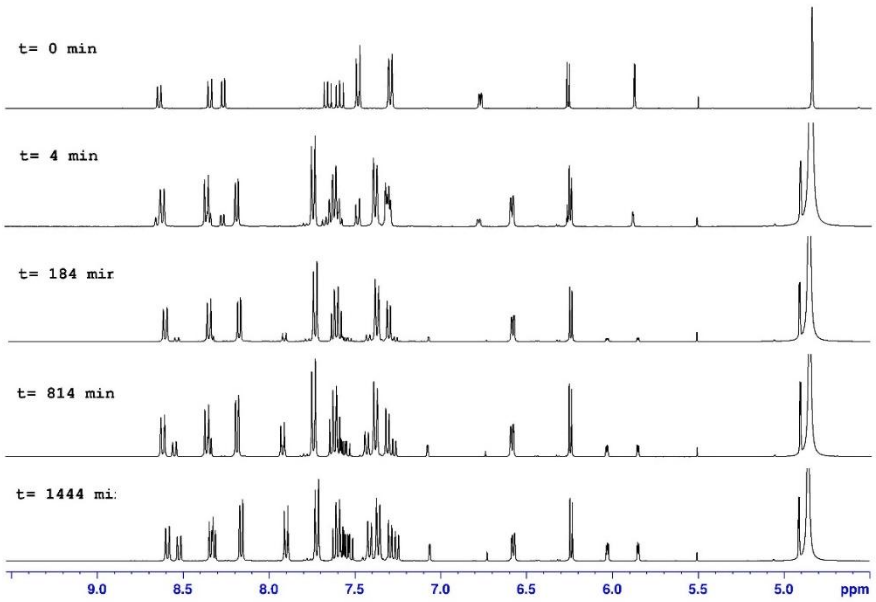

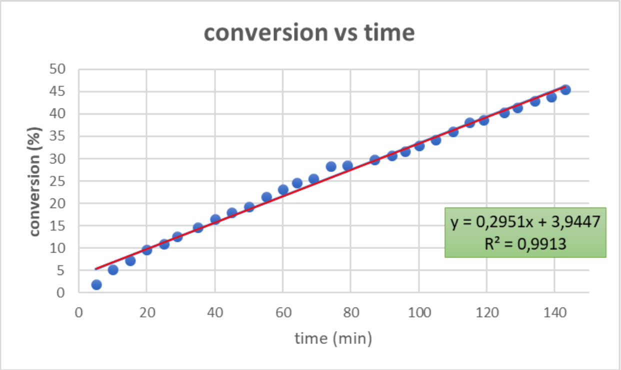

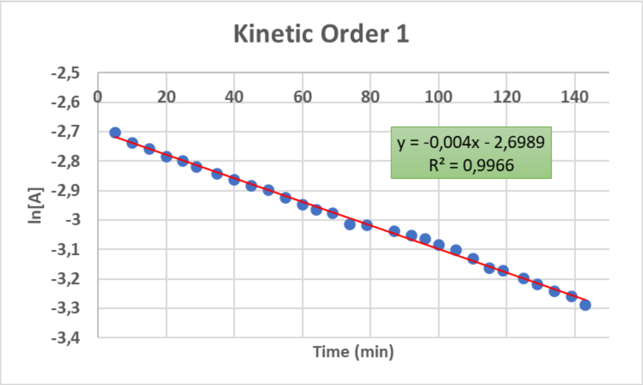

Figure S2. ^{1}H-NMR monitoring of the thiol-promoted fragmentation of thio-OND **7**. Determination of the kinetic order.

Compound 8

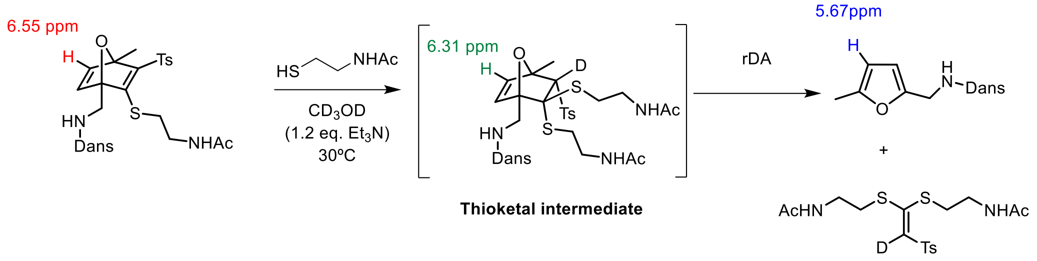

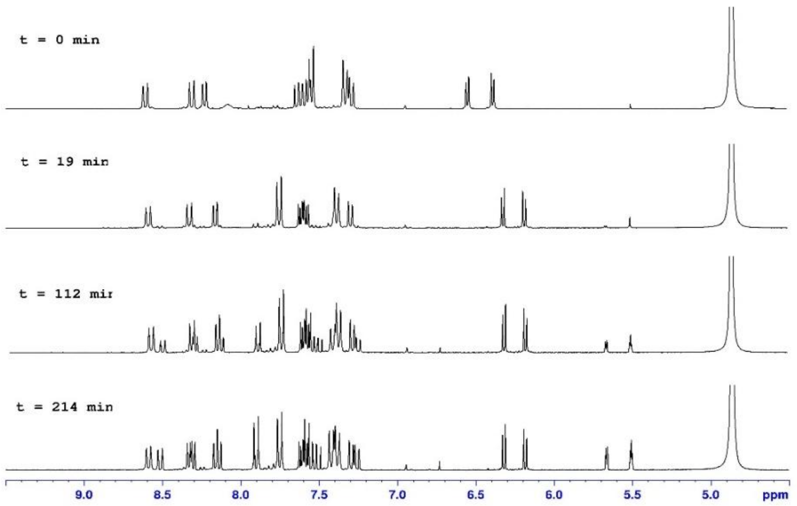

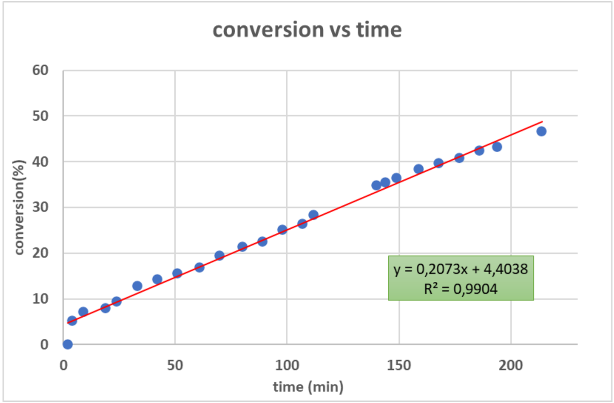

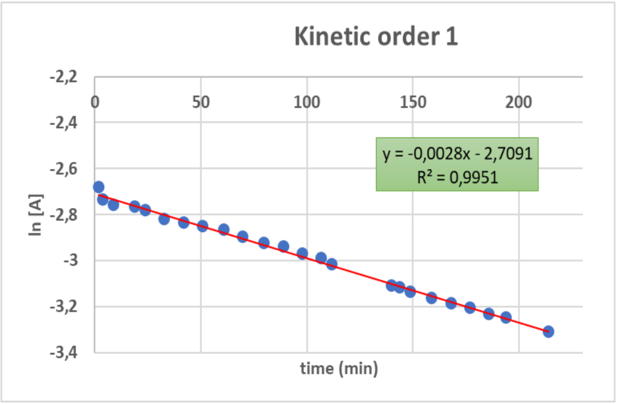

Figure S3. ^{1}H-NMR monitoring of the thiol-promoted fragmentation of thio-OND **8**. Determination of the kinetic order.

Compound 10

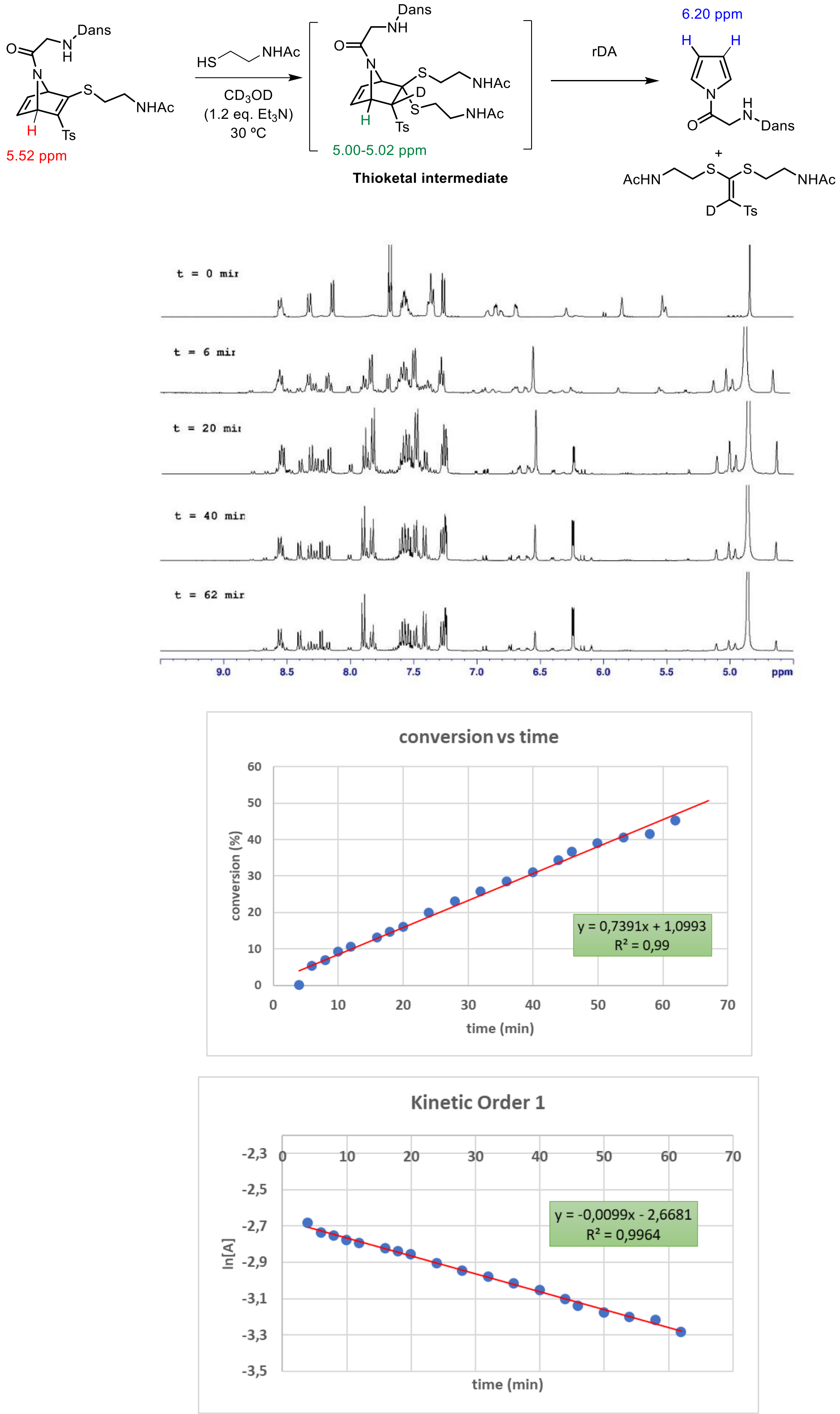

Figure S4. ^{1}H-NMR monitoring of the thiol-promoted fragmentation of thio-AND **10**. Determination of the kinetic order.

2.3 Study of the fragmentation of compound 10 with GSH in aqueous medium.

The thioketal intermediate is not detected by ^{1}H-NMR because the thio-Michael addition is much slower than the rDA fragmentation.

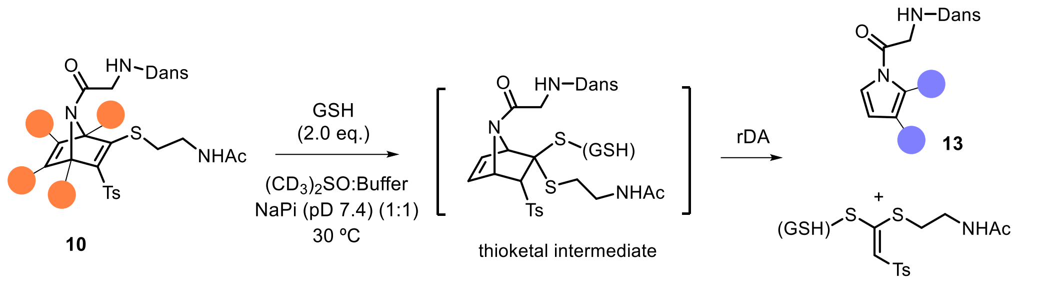

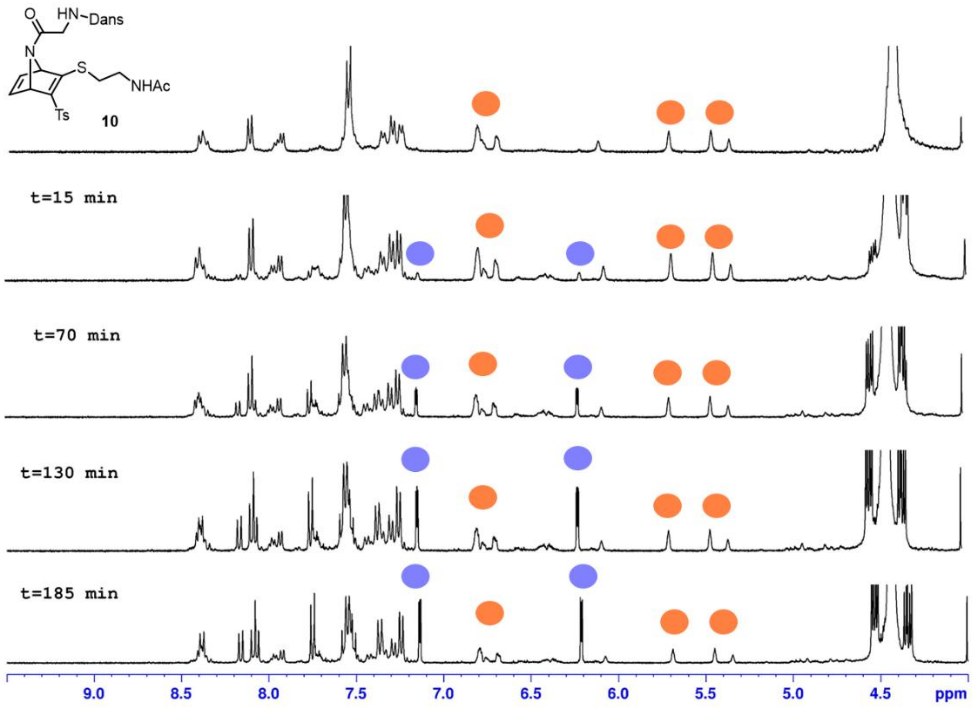

Figure S5. ^{1}H-NMR-monitoring of the GSH-promoted fragmentation of thio-AND **10** in aqueous medium.

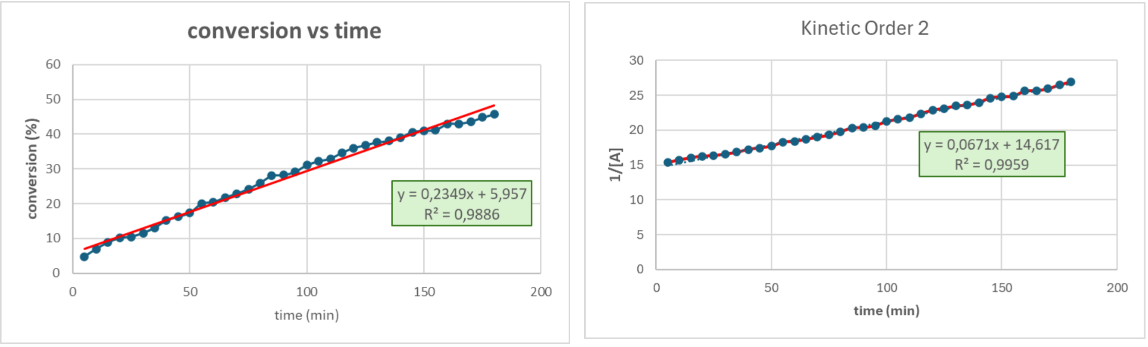

Figure S6. Determination of the kinetic order for the GSH-promoted fragmentation of thio-AND **10** in aqueous medium.

3. Fluorescence experiments.

3.1. General procedure for fluorescence measurements.

- Determination of emission curves: Stock solutions (10 mM in DMSO) and subsequent dilutions (200 µM in DMSO) of each compound were prepared. 50 µL of each compound (200 µM) was mixed with 50 µL of buffer (NaPi, 10 mM, 7.4 and 8.2) to achieve a final concentration of 100 µM in DMSO/Buffer 1:1. Emission spectrum of each solution was recorded in a Varioskan Lux ThermoFisher Scientific at 37 ºC, at a fixed excitation wavelength (334 nm). Each spectrum was recorded from 400 nm to 750 nm. Each spectrum was measured in triplicate (all these measurements were carried out in a greiner Black 96 well plate).
- Monitoring of the GSH-promoted HND fragmentation: Stock solutions of the corresponding HND (200 µM) in DMSO and a solution of GSH in buffer (NaPi, 50 mM, pH 8.2 or 7.4) (300, 450 or 750 µM) were prepared (GSH solutions were freshly prepared and handled in ice). Equal volumes (50 µL) of each solution were mixed to achieve a final HND concentration of 100 µM in DMSO:Buffer 1:1. The increase in fluorescence emission at 550 nm was monitored over time at 37 °C. Each measurement was performed in triplicates.

3.2. Comparative fluorescence emission plots of HNDs and related derivatives at different pHs (7.4 and 8.2).

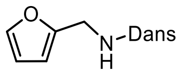

11

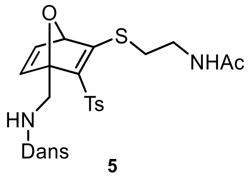

5

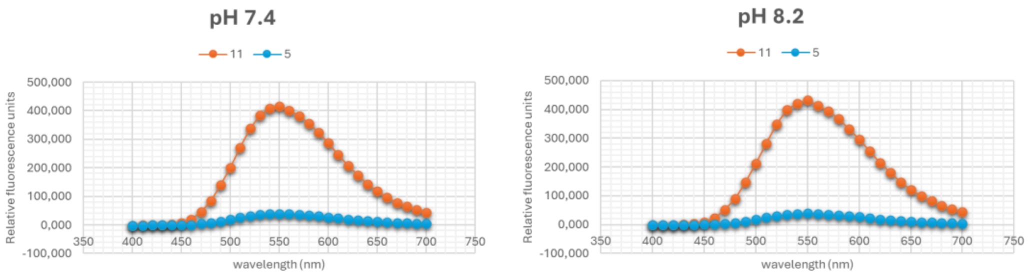

Figure S7. Comparative emission plots of compounds **5** and **11** at pH 7.4 and 8.2.

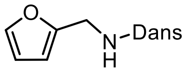

11

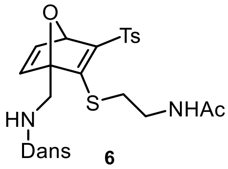

6

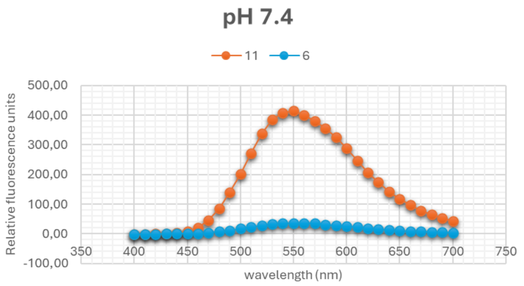

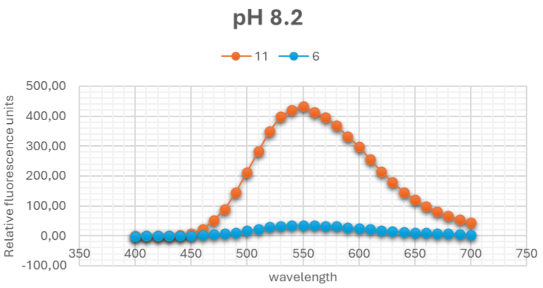

Figure S8. Comparative emission plots of compounds **6** and **11** at pH 7.4 and 8.2.

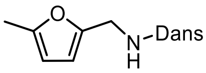

12

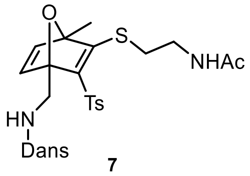

7

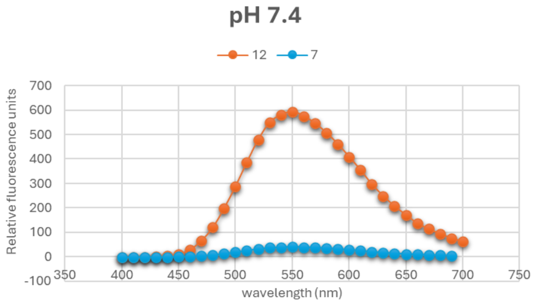

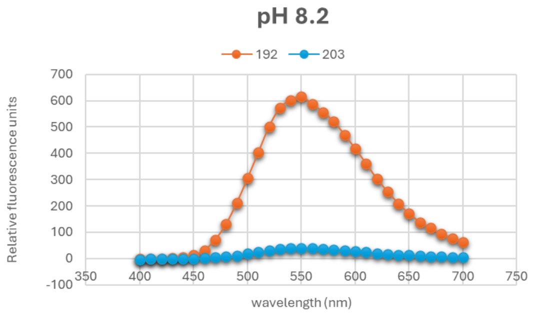

Figure S9. Comparative emission plots of compounds **7** and **12** at pH 7.4 and 8.2.

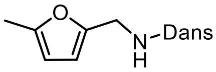

12

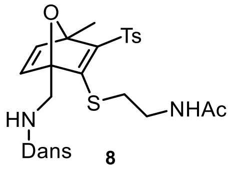

8

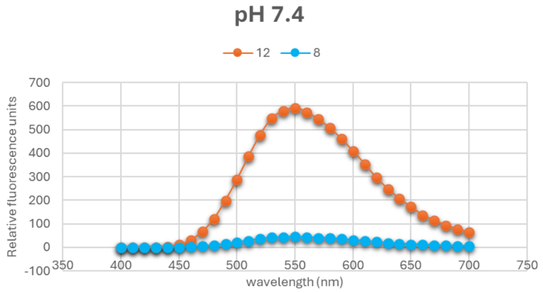

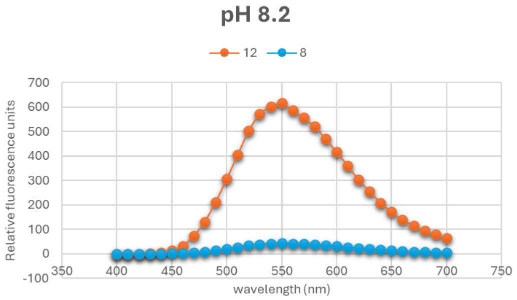

Figure S10. Comparative emission plots of compounds **8** and **12** at pH 7.4 and 8.2.

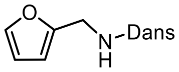

11

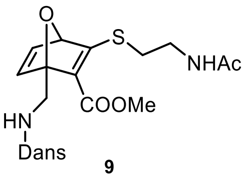

9

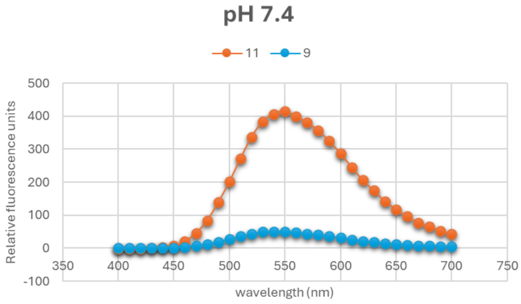

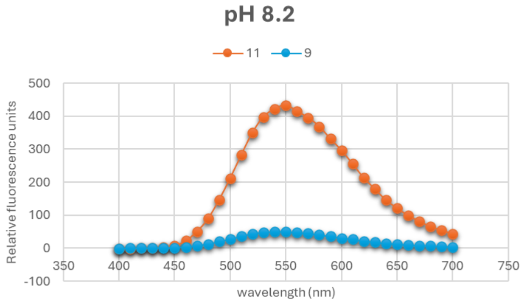

Figure S11. Comparative emission plots of compounds **9** and **11** at pH 7.4 and 8.2.

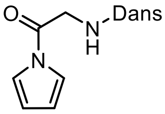

13

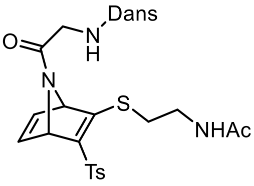

10

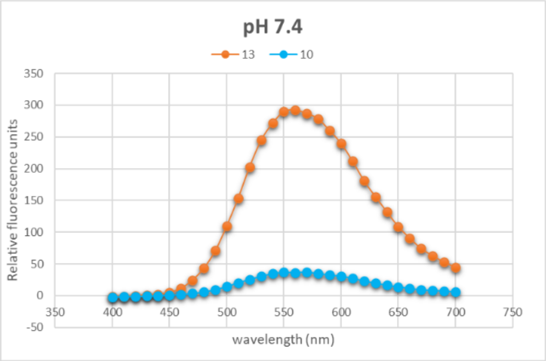

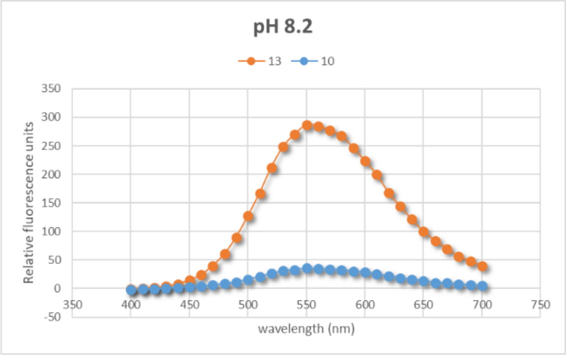

Figure S12. Comparative emission plots of compounds **10** and **13** at pH 7.4 and 8.2.

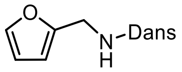

11

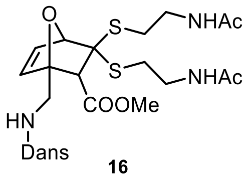

16

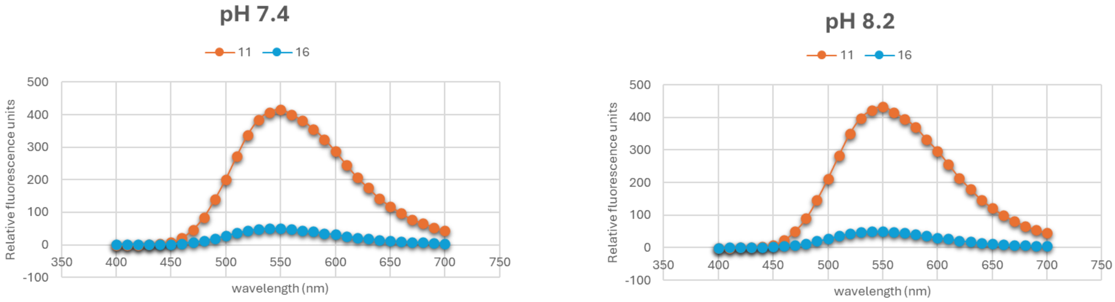

Figure S13. Comparative emission plots of compounds **16** and **11** at pH 7.4 and 8.2.

3.3. Monitoring the GSH-Promoted Fragmentation of HND (5 and 10) at Different pH Values and GSH Concentrations

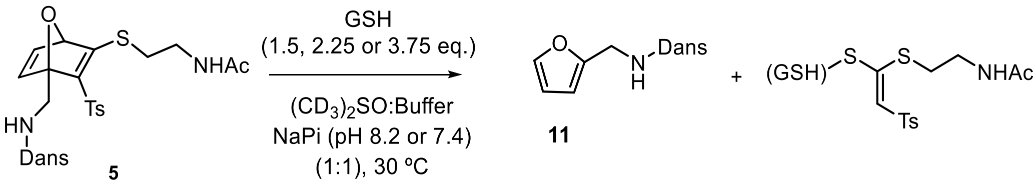

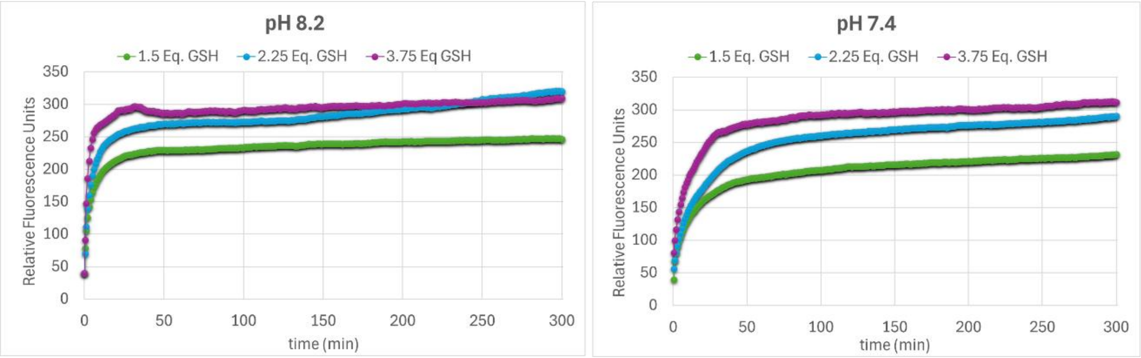

Figure S14. Fluorescence intensity *vs*. time for compound **5** at different GSH concentrations at pH 8.2 and 7.4.

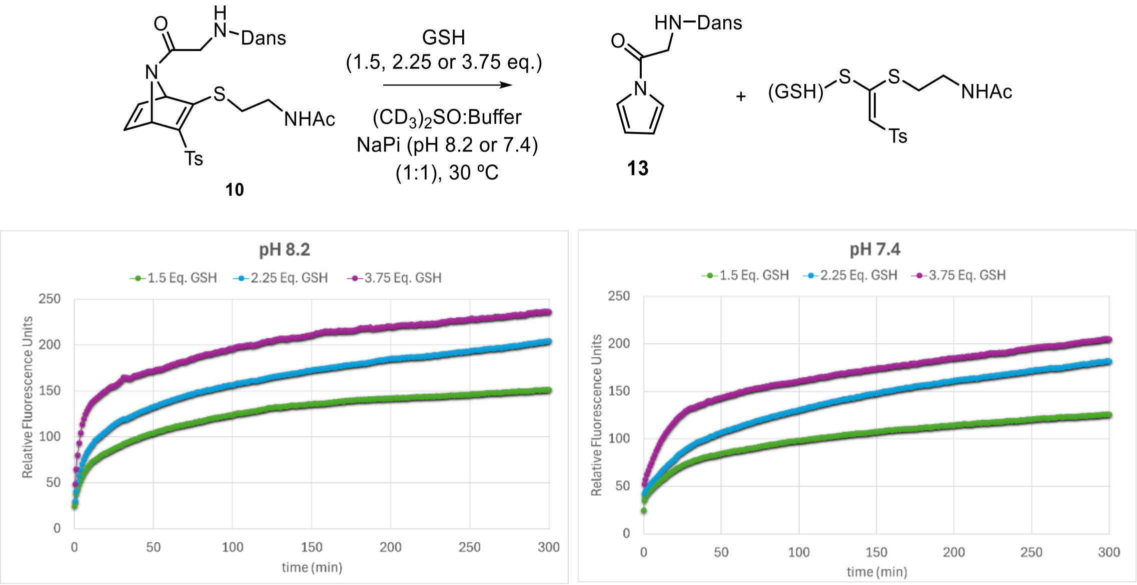

Figure S15. Fluorescence intensity *vs*. time for compound **10** at different GSH concentrations at pH 8.2 and 7.4.

3.4. Summary table.

Compound	Structure[a]	K_{obs} (min^{-1})[b]	$t_{1/2}$ (h)	Fluorescence increase (pH 8.2)[c]	Fluorescence increase (pH 7.4)[c]	Maximum fluorescence (pH 8.2)[d]	Maximum fluorescence (pH 7.4)[d]
**5**	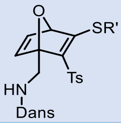	2.5×10^{-4}	19	122.6	69.1	39.6	39.6
**6**	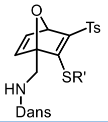	3.3×10^{-4}	26	131.2	92.3	35.9	35.9
**7**	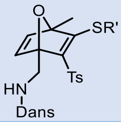	4.0×10^{-3}	3.1	132.5	113.0	40.4	39.5
**8**	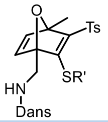	2.8×10^{-3}	3.7	149.3	112.8	45.2	42.3
**9**	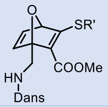	--	--	--	--	37.4	35.6
**10**	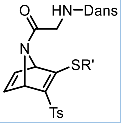	9.9×10^{-3}	1.2	58.9	43.5	56.9	50.8

Table S1. a) R'=CH_2CH_2NHAc; b) First-order rate constant for the fragmentation reaction; c) Increase in fluorescence intensity (relative units; λ_{em} = 550 nm, λ_{ex} = 334 nm) observed 20 min after the addition of 300 µM glutathione to the compound, relative to the emission intensity before addition, at 37 °C; d) Maximum fluorescence intensity (relative units) of dansylated HNDs (λ_{ex}= 334 nm, λ_{em}= 550 nm).

4. Cell imaging experiments.

U87-MG cells were routinely passaged according to the recommendations and seeded one day before the experiment at a density of 100,000 cells / well, in µ-Slide 8 Well slide (ibidi Cat.No: 80826) in 200 µL of media. On the day of the experiment, the cells were treated with the corresponding HND or vehicle (DMSO) control. After selected incubation period the cells were washed (PBS, pH 7.4, 3×), fixed with 4% paraformaldehyde (20 min, room temperature), washed again (PBS, pH 7.4, 3×), and the membrane was stained by treating with Wheat Germ Agglutinin – Alexa Fluor 647 conjugate (InvitrogenTM catalogue number W32466) according to manufacturer instructions, washed 3 times (PBS, pH 7.4, 3×), and FluoroBrite™ DMEM media (200 µL, Thermo Fisher A1896701) was added before proceeding to imaging.

Fixed cells were then imaged using a Leica DMi8 confocal microscope with a 40× oil immersion objective. For each sample, at least 3 images were taken at randomly assigned fields, at least three independent biological replicates were performed in each experiment unless stated otherwise. Images were acquired using channels standard DAPI, (λ_{ex} = 405 nm) and Alexa Fluor 647 (λ_{ex} = 647 nm) filter cubes, including brightfield image acquisition. For quantitative analysis, raw images were imported using "default" colour mode into Fiji ImageJ2 (version 2.14.0/1.54f). The mean fluorescence per cell were measured by creating binary image from cell membrane (channel 647), creating single cells using *watershed* function, selecting regions of interest (ROI) above 100 μm^2 and then using *measure* function on 405 channel. All resultant values were normalised to the median of vehicle control sample (Median (MFI, DMSO) = 1.0) for each biological replicate. For comparative pictorial purposes only, the maximum colour for Channel 405 nm was manually set to a value of 30 for every image using the colour balance toll, while Channel 647 nm was adjusted automatically.

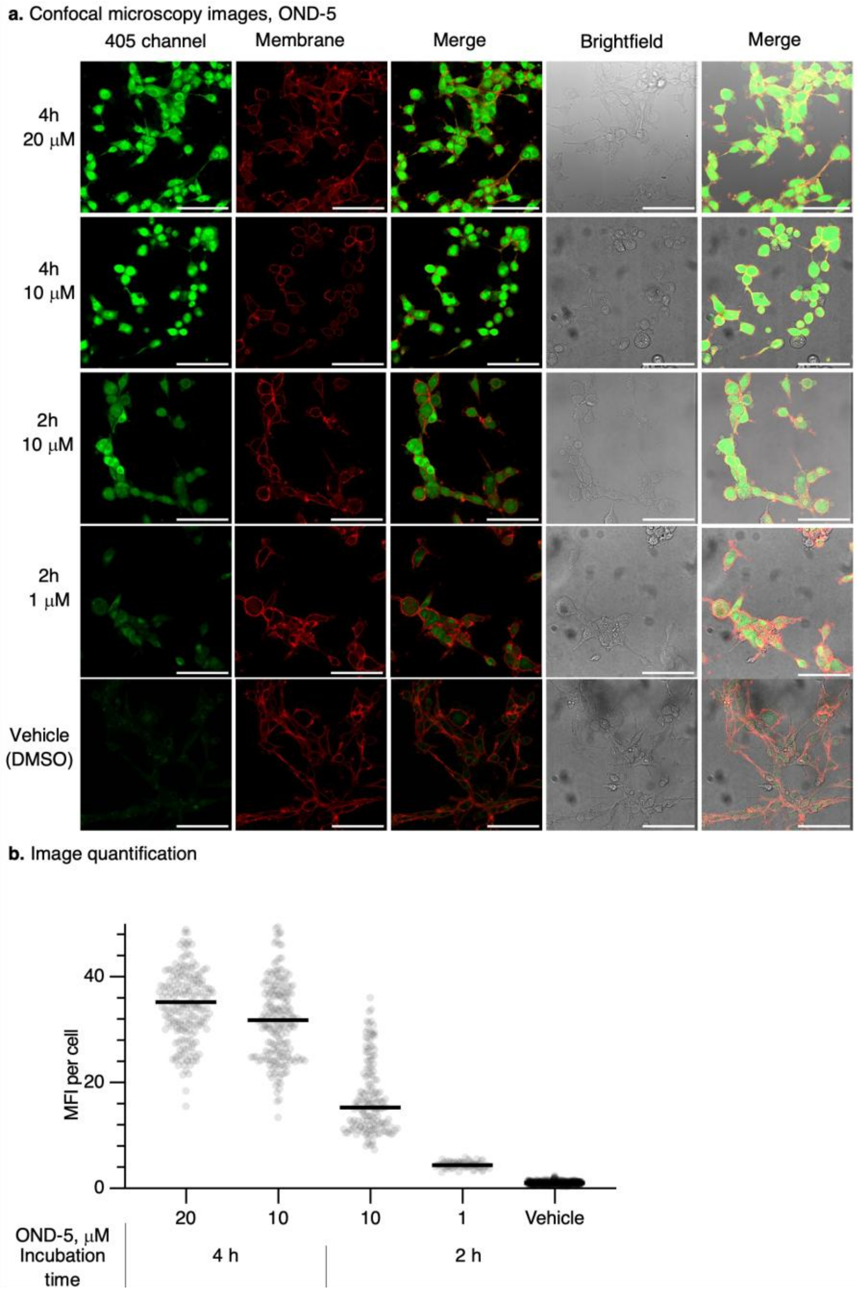

Figure S16 a. Confocal microscopy images of U87-MG cells stained with Wheat Germ Agglutinin – Alexa Fluor 647 (cell membrane, red; λ_{ex} = 650 nm, λ_{em} = 665 nm) and after incubation with OND-**5** (20 – 1 µM) for 4 or 2 h at 37 °C. The dansyl fluorescence corresponding to OND activation is shown in green (λ_{ex} = 405 nm, λ_{em} = 550 nm). The white scale bar represents 100 µm. **b.** Turn-on fluorescence quantification (image-wise ratio to DMSO vehicle control channel fluorescence) suggested significant OND-**5** fluorescence in various concentrations. Each dot represents a single cell; data sets combine independent experiments.

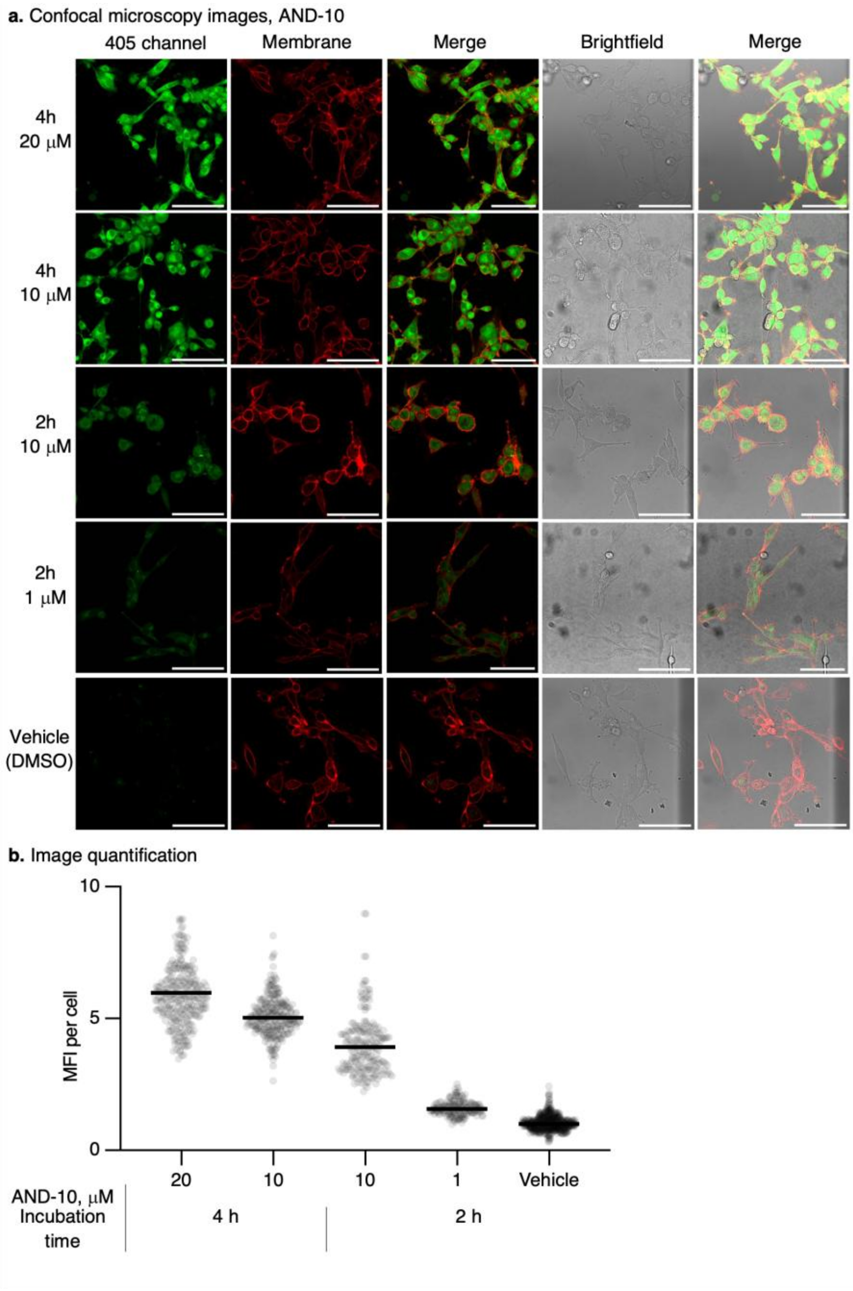

Figure S17 a. Confocal microscopy images of U87-MG cells stained with Wheat Germ Agglutinin – Alexa Fluor 647 (cell membrane, red; λ_{ex} = 650 nm, λ_{em} = 665 nm) and after incubation with AND-**10** (20 – 1 μM) for 4 or 2 h at 37 °C. The dansyl fluorescence corresponding to AND activation is shown in green (λ_{ex} = 405 nm, λ_{em} = 550 nm). The white scale bar represents 100 μm. **b.** Turn-on fluorescence quantification (image-wise ratio to DMSO vehicle control channel fluorescence) suggested significant AND-**10** fluorescence in various concentrations. Each dot represents a single cell; data sets combine independent experiments.

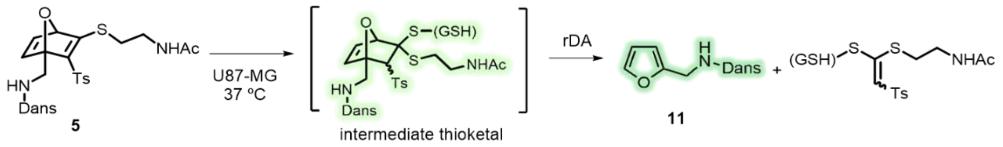

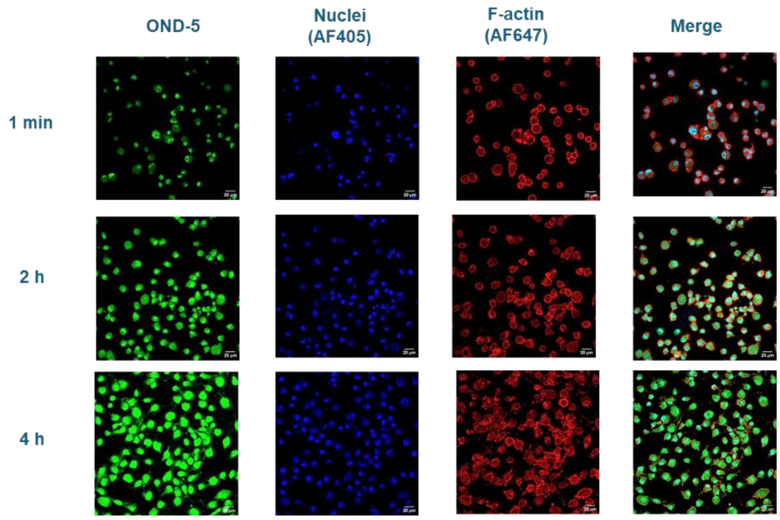

Figure S18. Fluorescence microscopy images of U87-MG cells stained with Phalloidin-iFluor® 647 (F-actin, red; λ_{ex} = 650 nm, λ_{em} = 665 nm) and DAPI (nucleus, blue; λ_{ex} = 358 nm, λ_{em} = 460 nm) after incubation with OND **5** (20 µM) for 1 min, 2 h, and 4 h at 37 °C. The dansyl fluorescence corresponding to OND activation is shown in green (λ_{ex} = 334 nm, λ_{em} = 550 nm).

5. Cell viability assays

Cells were seeded in Corning Costar 96-well clear flat-bottom plates at 20,000 cells per well in 150 µL of media one day before the experiment. The following day, compounds of interest were diluted to 4× of desired concentrations in 50 µL and added to the seeded cells. Cells were incubated for 4 or 24 hours before the viability reagent was added (CellTiter-Blue® Cell Viability Assay G8080), and the fluorescence readout was measured after another 1 hour incubation at 37°C (λ_{ex} = 540 nm, λ_{em} = 590 nm) with Molecular Devices SpectraMax MiniMax 300 Imaging cytometer i3x. Cell viability% was calculated as 100% × F_{cells} / $F_{vehicle\_control}$. F refers to the fluorescence of a given well. 0% cell viability refers to control of 100 µM digitonin.

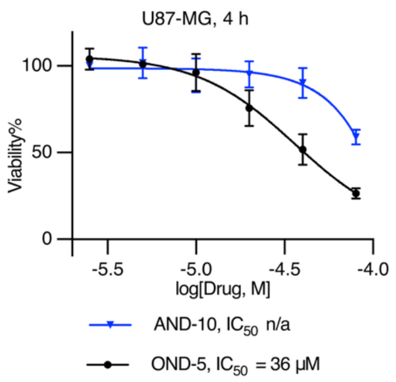

Figure S19. Cell viability of OND **5** and AND **10** towards U87-MG cell line up. Error bars represent ± standard deviation (n = 3). The experiment was repeated three times for each compound and cell line using CellTiter blue™ assay. Log (10 µM) = -5.0. Viability of 100% refers to the viability of the cells in the control wells. Each experiment had a cytotoxic drug control (digitonin).

6. ^{1}H- and ^{13}C-NMR spectra for new compounds.

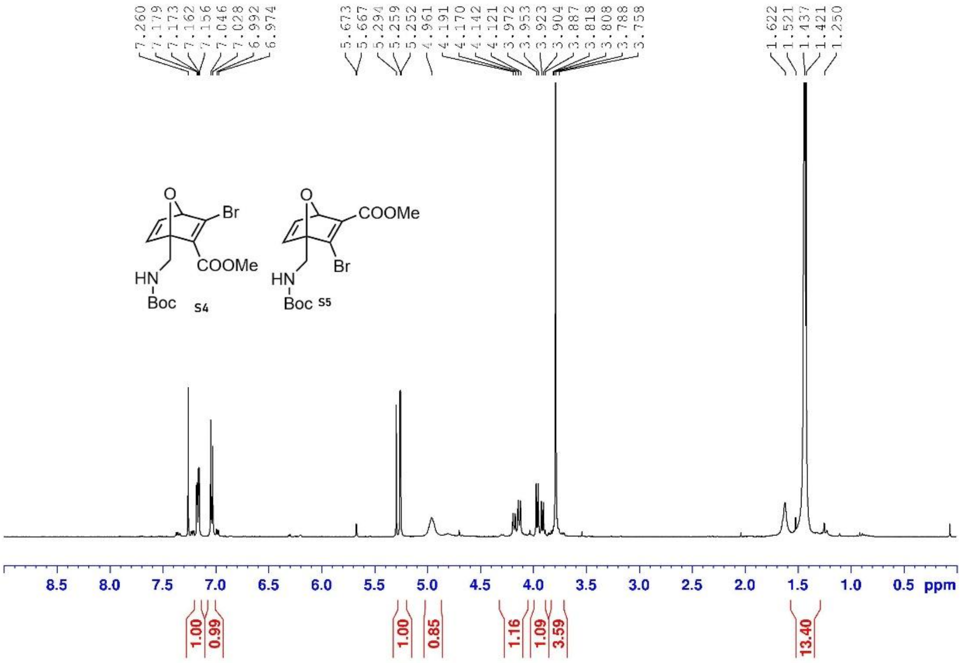

^{1}H-NMR ($CDCl_3$, 300 MHz) of the mixture of compounds **S4** and **S5** (*ratio **S4**:**S5** = 10:1*)

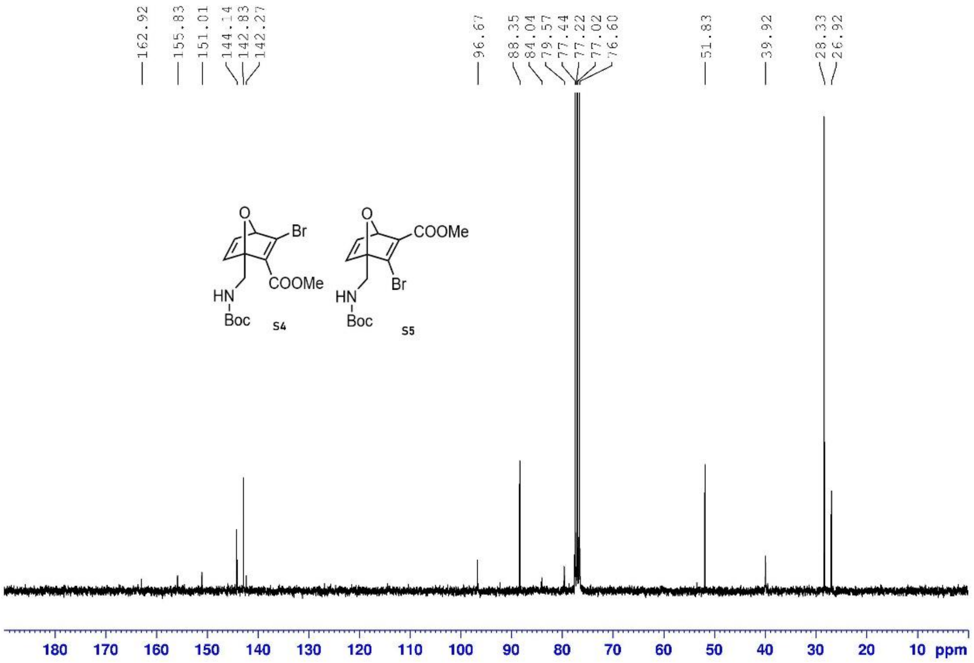

^{13}C-NMR ($CDCl_3$, 75 MHz) of the mixture of compounds **S4** and **S5**

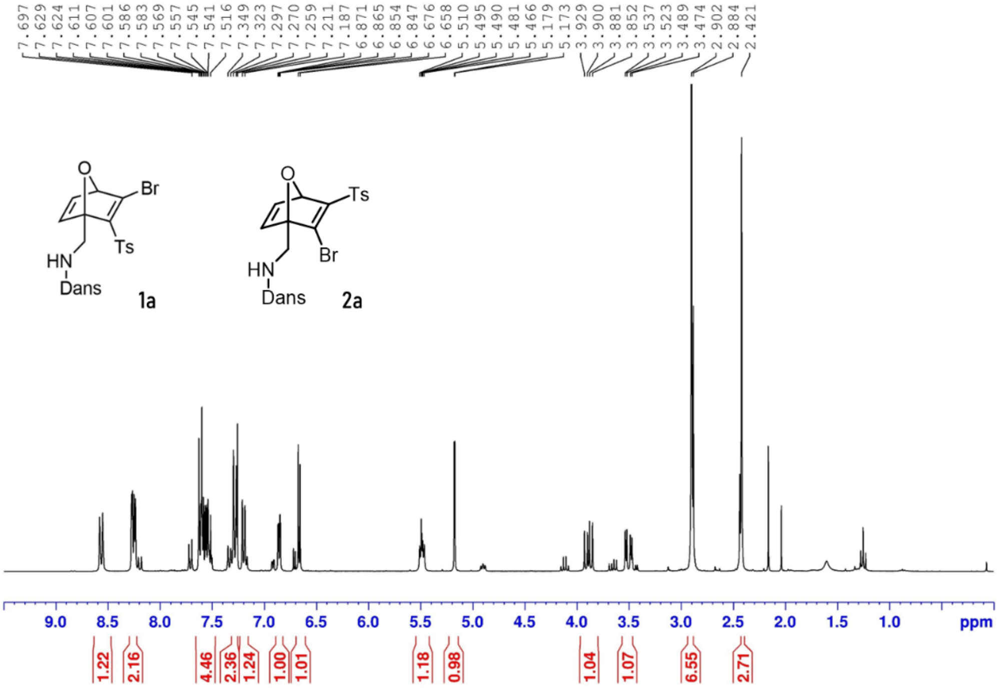

^{1}H-NMR ($CDCl_3$, 300 MHz) of the mixture of compounds **1a** and **2a** (*ratio **1a**:**2a** = 5:1*)

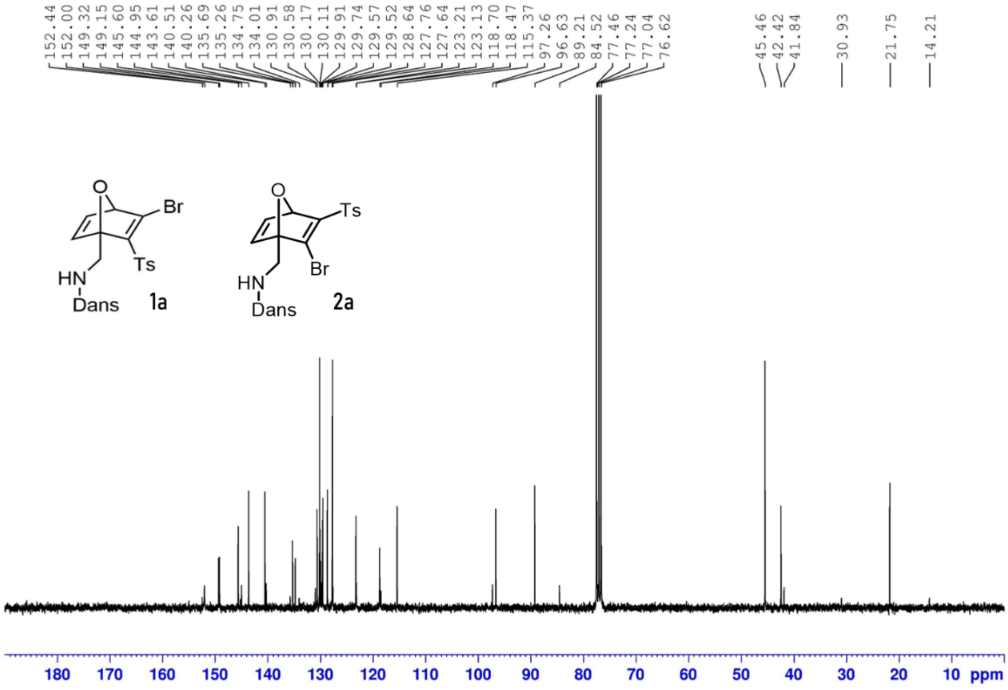

^{13}C-NMR ($CDCl_3$, 75 MHz) of the mixture of compounds **1a** and **2a**

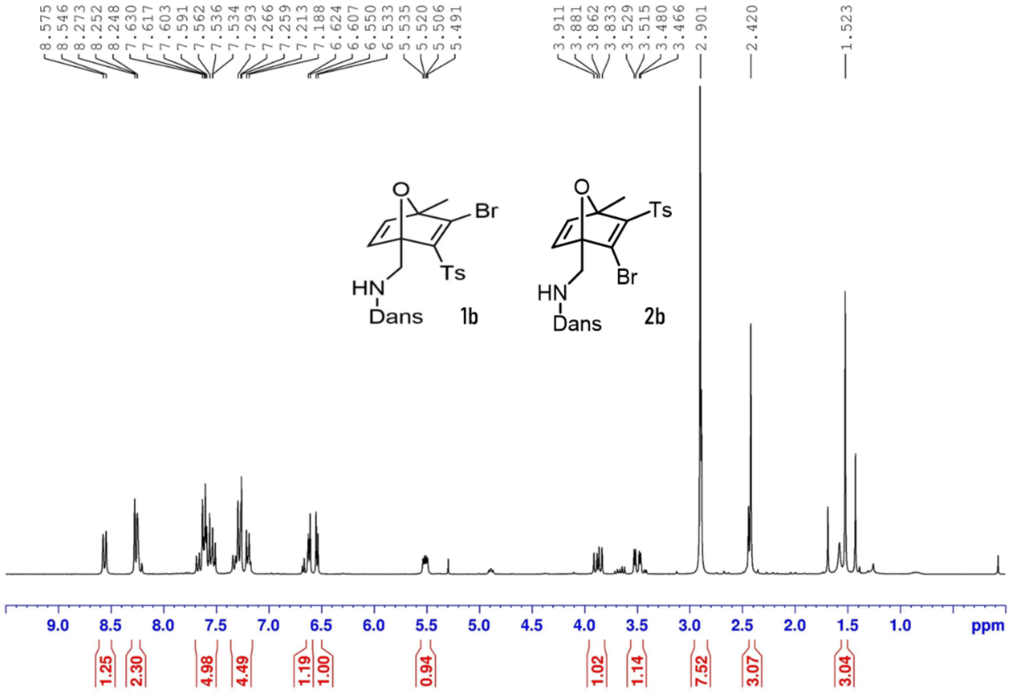

^{1}H-NMR ($CDCl_3$, 300 MHz) of the mixture of compounds **1b** and **2b** (*ratio **1b**:**2b** = 5:1*)

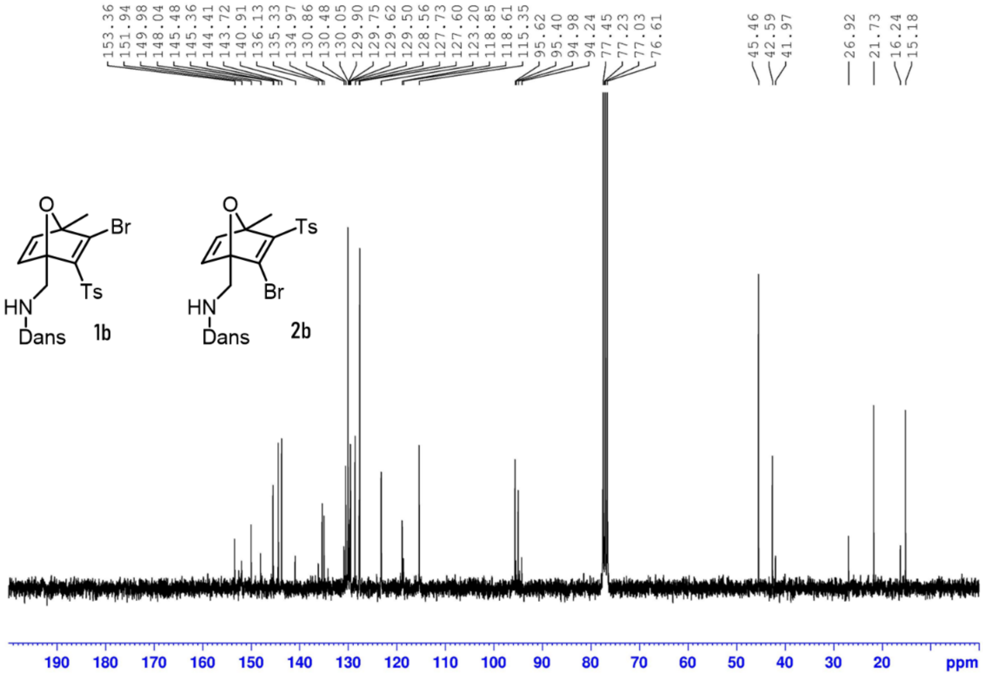

^{13}C-NMR ($CDCl_3$, 75 MHz) of the mixture of compounds **1b** and **2b**

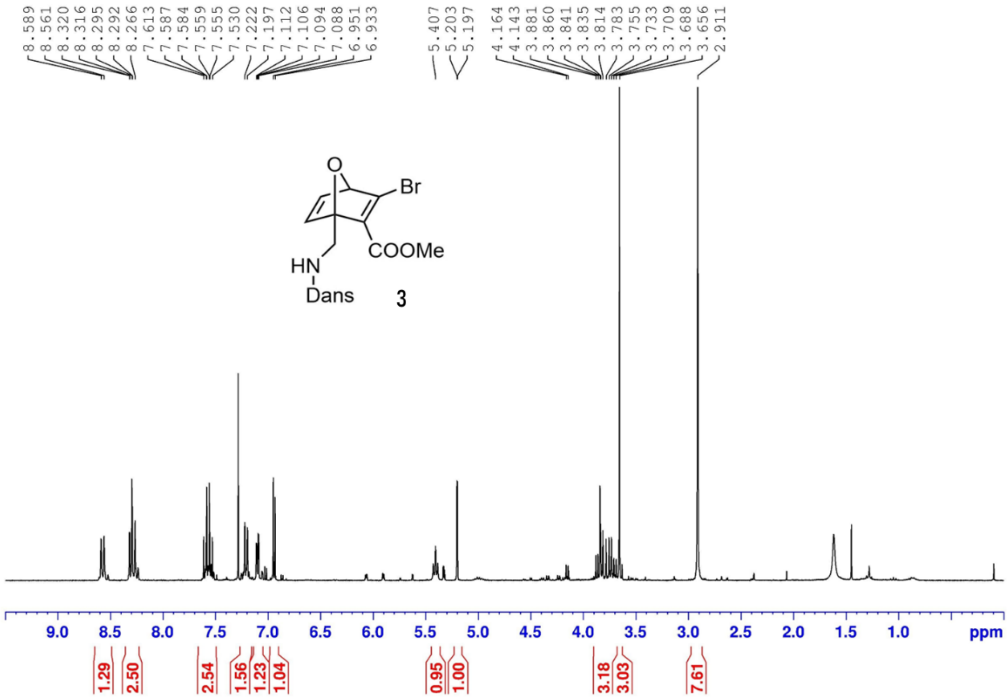

^{1}H-NMR ($CDCl_3$, 300 MHz) of compound **3**

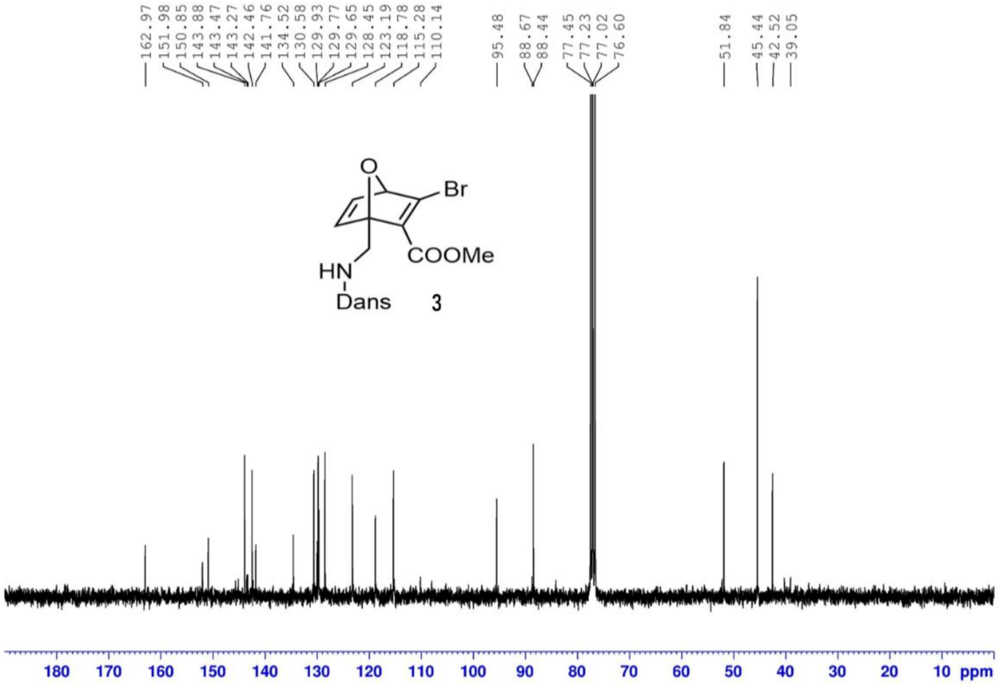

^{13}C-NMR ($CDCl_3$, 75 MHz) of compound **3**

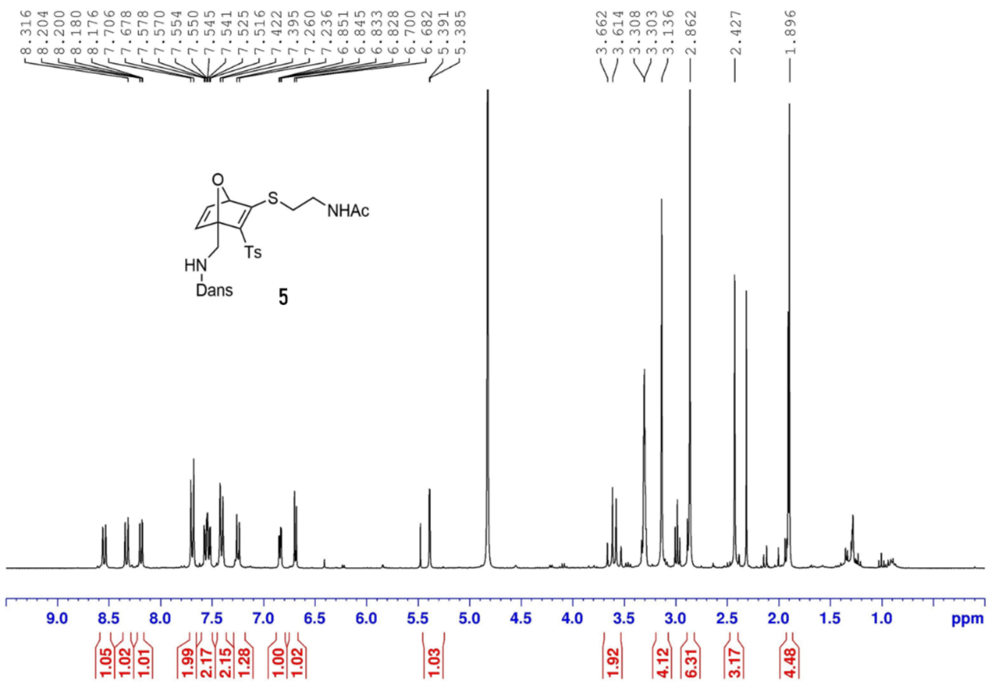

^{1}H-NMR (CD_3OD, 300 MHz) of compound **5**

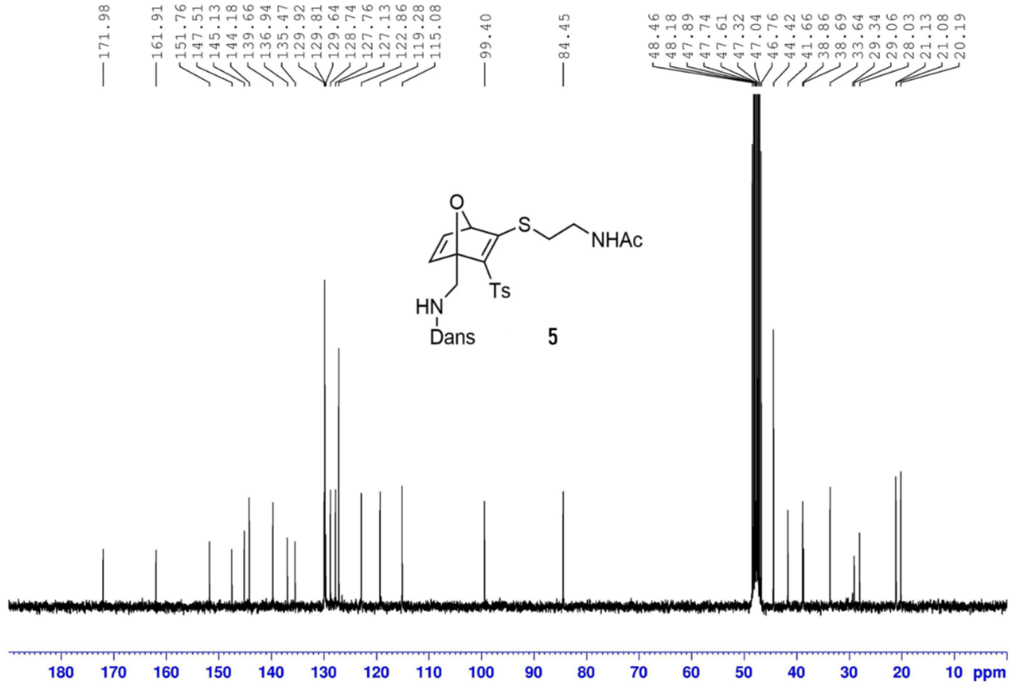

^{13}C-NMR (CD_3OD, 75 MHz) of compound **5**

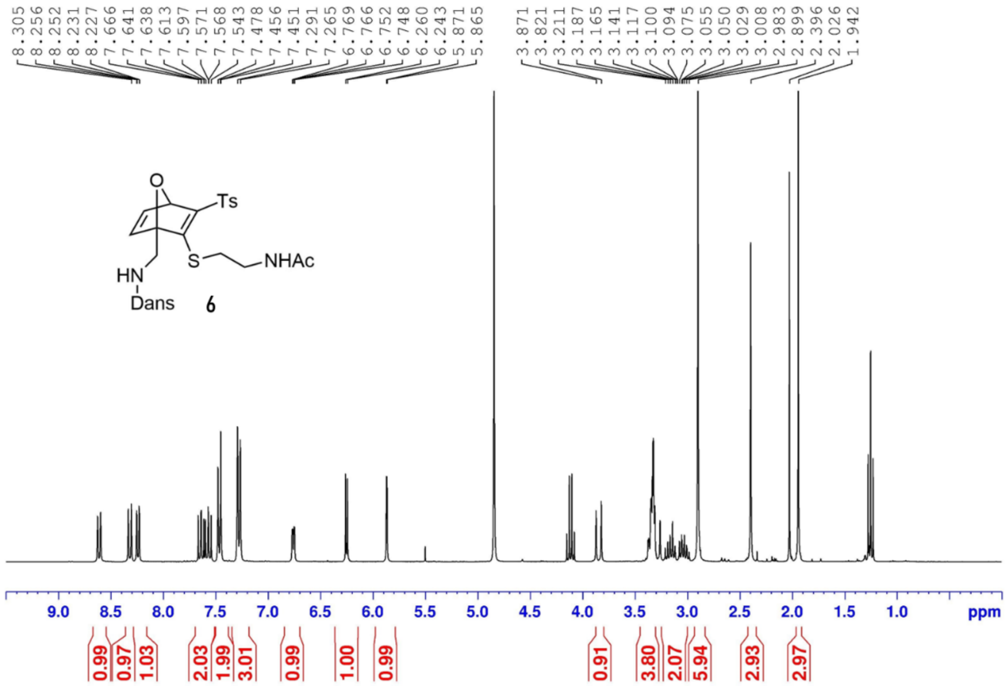

^{1}H-NMR (CD$_3$OD, 300 MHz) of compound **6**

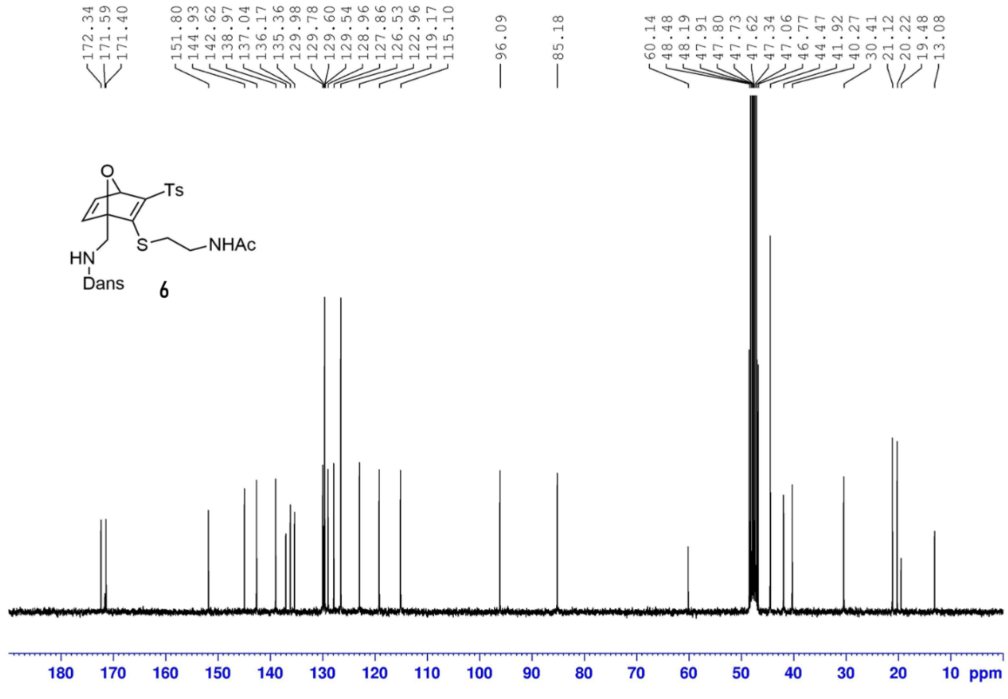

^{13}C-NMR (CD$_3$OD, 75 MHz) of compound **6**

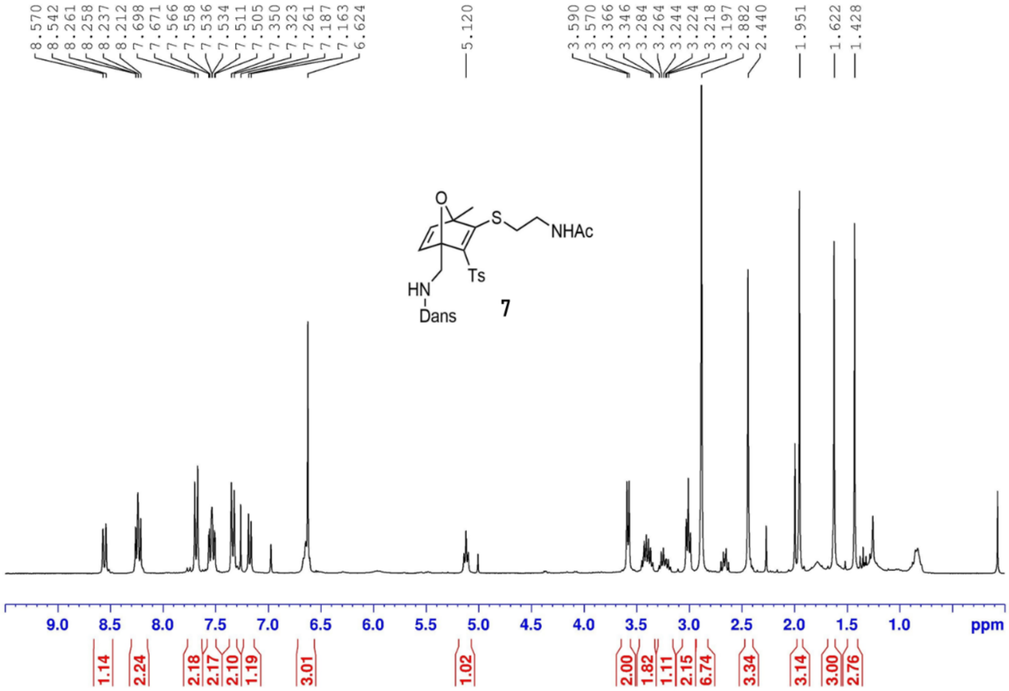

^{1}H-NMR ($CDCl_3$, 300 MHz) of compound **7**

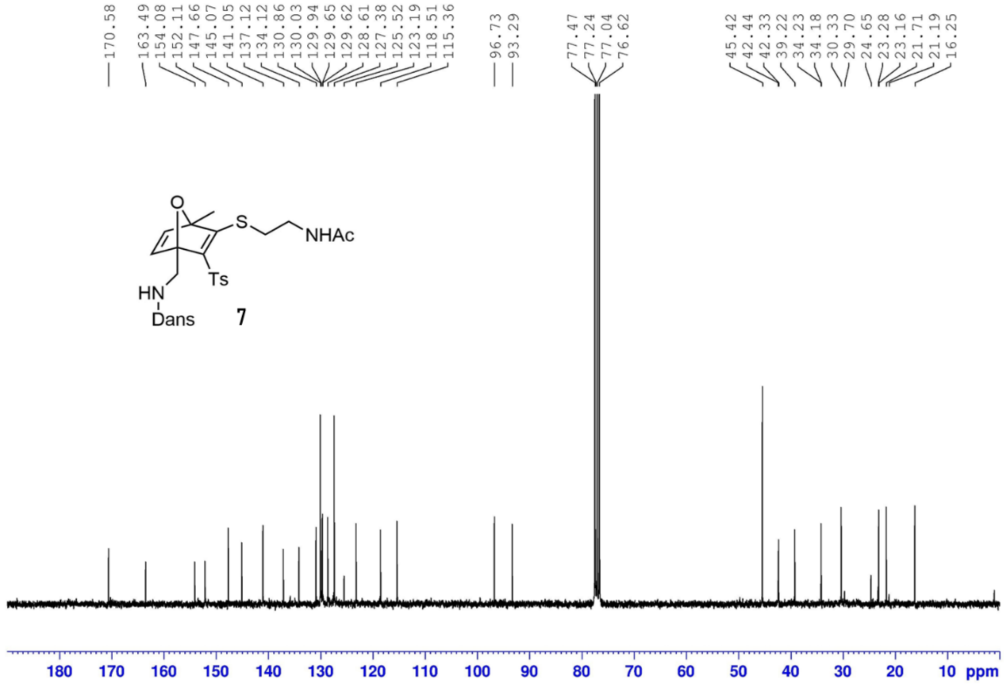

^{13}C-NMR ($CDCl_3$, 75 MHz) of compound **7**

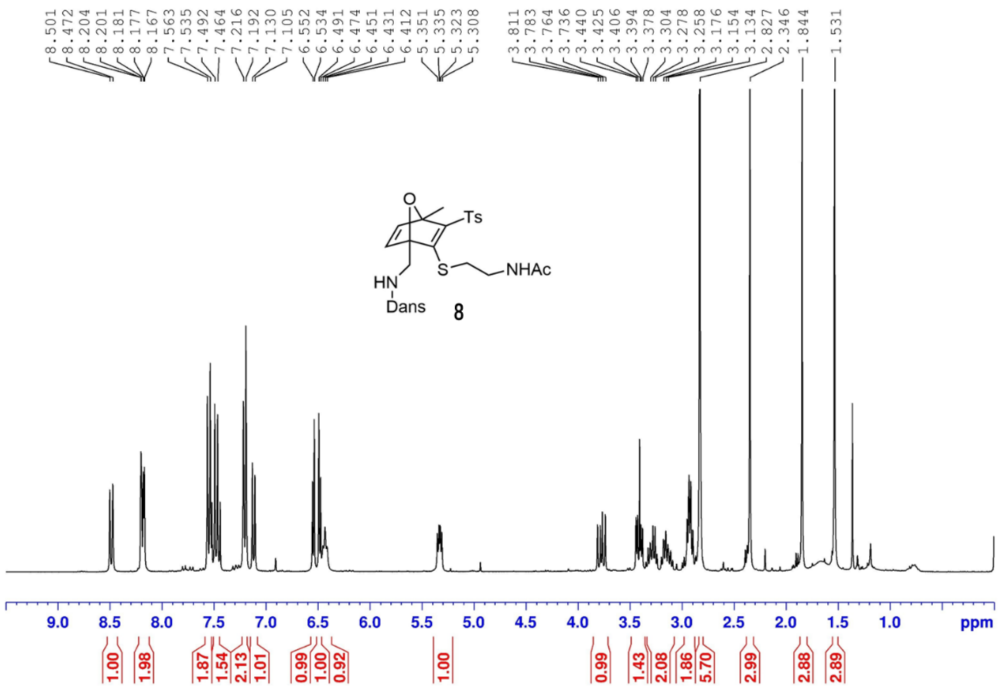

^{1}H-NMR (CDCl$_3$, 300 MHz) of compound **8**

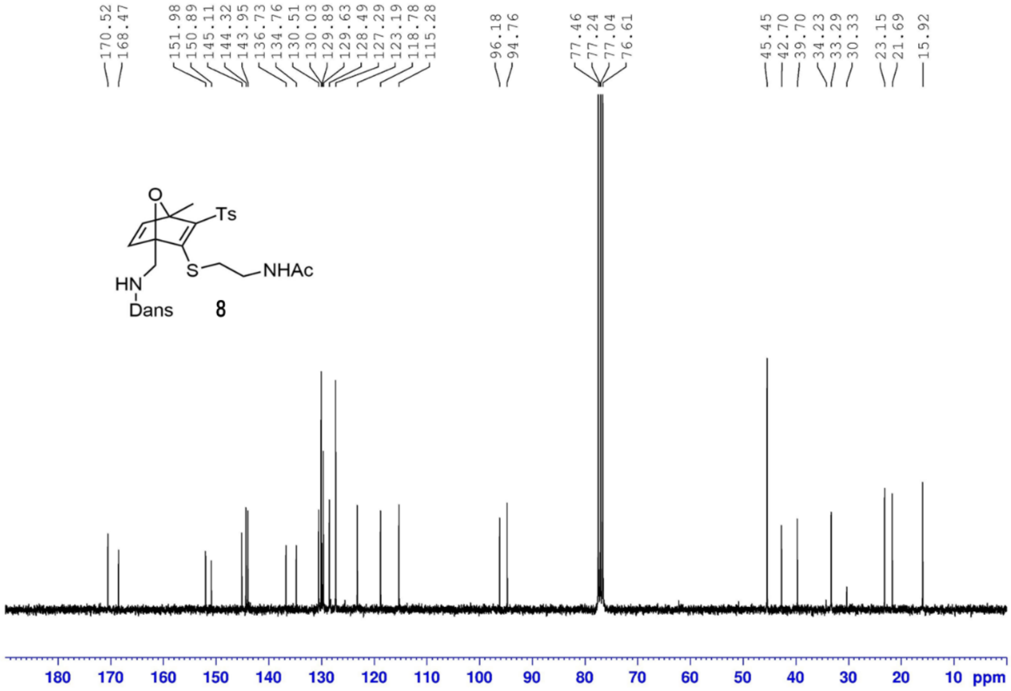

^{13}C-NMR (CDCl$_3$, 75 MHz) of compound **8**

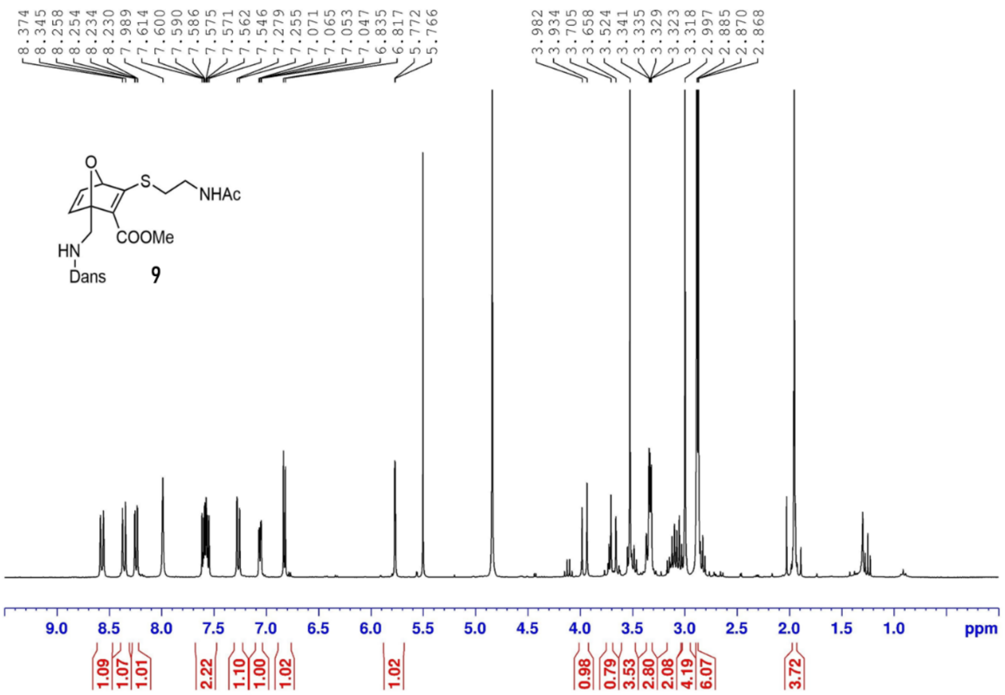

^{1}H-NMR (CD_3OD, 300 MHz) of compound **9**

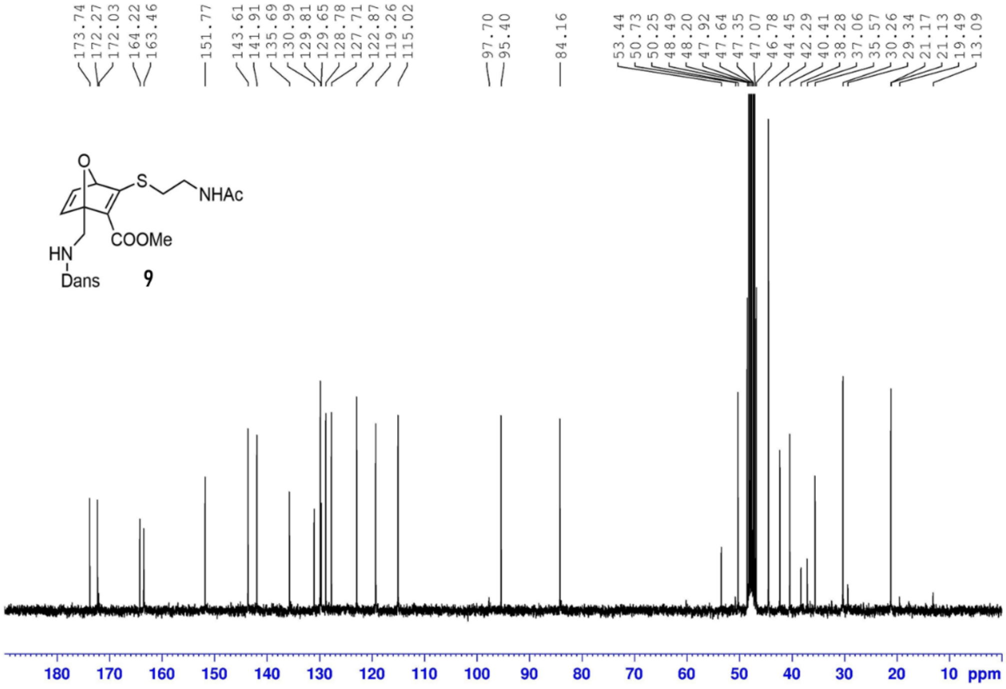

^{13}C-NMR (CD_3OD, 75 MHz) of compound **9**

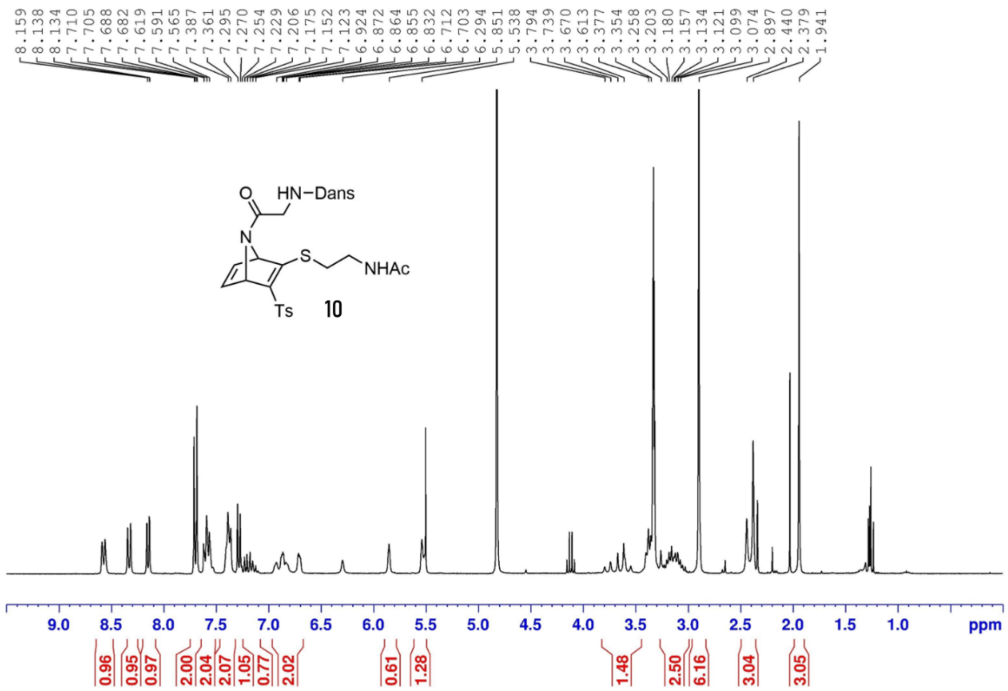

^{1}H-NMR (CD_3OD, 300 MHz) of compound **10**

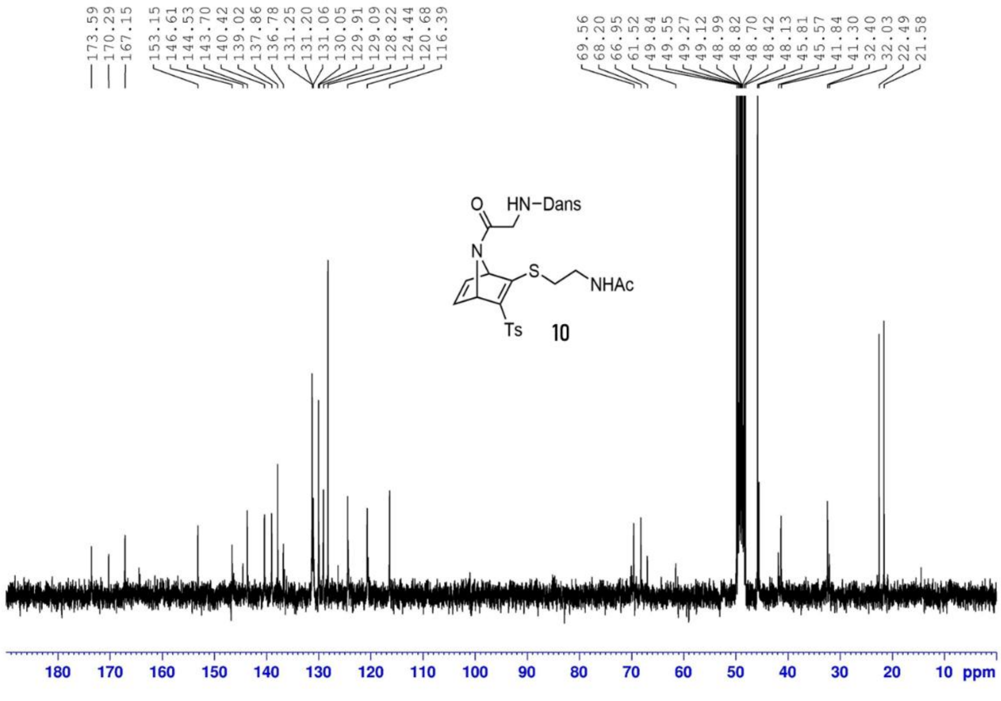

^{13}C-NMR (CD_3OD, 75 MHz) of compound **10**

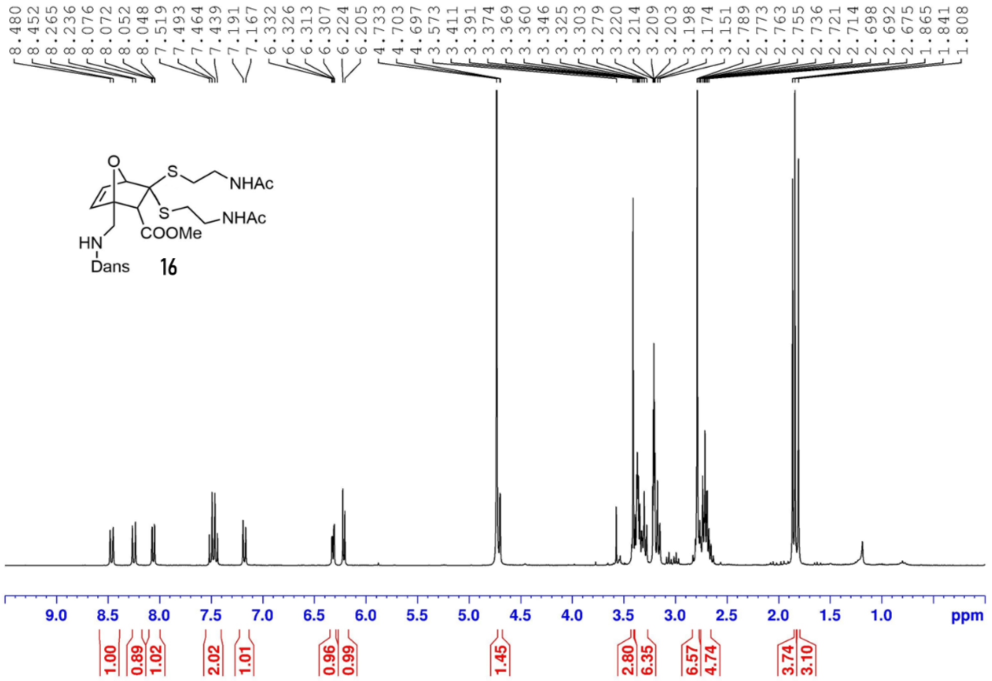

^{1}H-NMR (CD_3OD, 300 MHz) of compound **16**

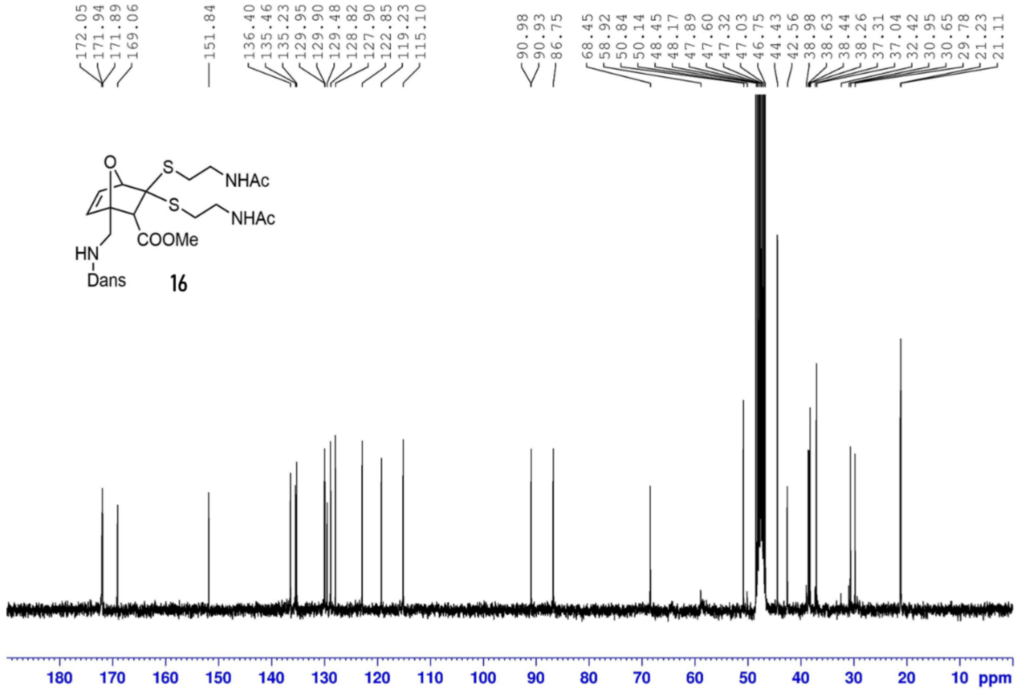

^{13}C-NMR (CD_3OD, 75MHz) of compound **16**

6. References

1. Hong, V.; Kislukhin, A. A.; Finn, M.G. Thiol-Selective Fluorogenic Probes for Labeling and Release. *J. Am. Chem. Soc.* **2009**, *131*, 9986-9994.
2. Zhang, C.; Ballay II, C. J.; Trudell, M. L. 2-Bromoethynyl Aryl Sulfones as Versatile Dienophiles: A Formal Synthesis of Epibatidine. *J. Chem. Soc., Perkin Trans*. **1999**, *1*, 675−676.
3. Carranza, M.; Carmona, A. T.; Maya, Gil de Montes, D.; Vasco, A.; Bernardes, G.; Moreno-Vargas, A. J. [2.2.1]Heterobicyclic bromovinyl sulfones for thiol-triggered strategies in linker chemistry: aza- vs oxanorbornadienic systems. *Bioconj. Chem*. **2025**, *36*, 2079−2089.
